# The miniaturized vacuum system for cold atom sensors based on the technology of passive vacuum

**DOI:** 10.1038/s41598-026-51873-5

**Published:** 2026-05-28

**Authors:** Wenfeng He, Mengxin Zheng, Zecheng Zhang, Nan Guo, Yiren Sun, Chenjie Zhai, Bin Wu, Xiaolong Wang, Qiang Lin

**Affiliations:** 1 Zhejiang Provincial Key Laboratory of Quantum Precision Measurement, Zhejiang University of Technology, Hangzhou, 310023 DC China; 2 School of Physics and Institute of Quantum Precision Measurement, Zhejiang University, Hangzhou, 310058 China

**Keywords:** Engineering, Optics and photonics, Physics

## Abstract

Over the past decade, the cold atom sensors have transitioned gradually from laboratory-based experiments to practical field applications. The miniaturization is of great importance and needs to be achieved for this transition, especially for the vacuum system for cold atom sensors. Typically, the vacuum system is complicated and usually composed of the vacuum chamber, the optical path for the laser beam, and the magnetic field coils. The improvement of size, weight, and power (SWaP) consumption of the vacuum system has become a challenge at present. In this paper, the passive vacuum technology is proposed and overviewed to simplify the vacuum system for cold atom sensors (VSCAS). Firstly, the materials of low helium-permeability are summarized. Secondly, the key components required for the realization of the passive vacuum system (PVS) are discussed in great detail, including the getter pump and atomic source. Then, the integration of the passive vacuum system with the mentioned components is summarized, and the evaluation methods of the performance of those vacuum systems have been analyzed. Moreover, we have carried out the preliminary designs and experiments of PVS, and present some experimental results to verify its feasibility.

## Introduction

**Fig. 1:**
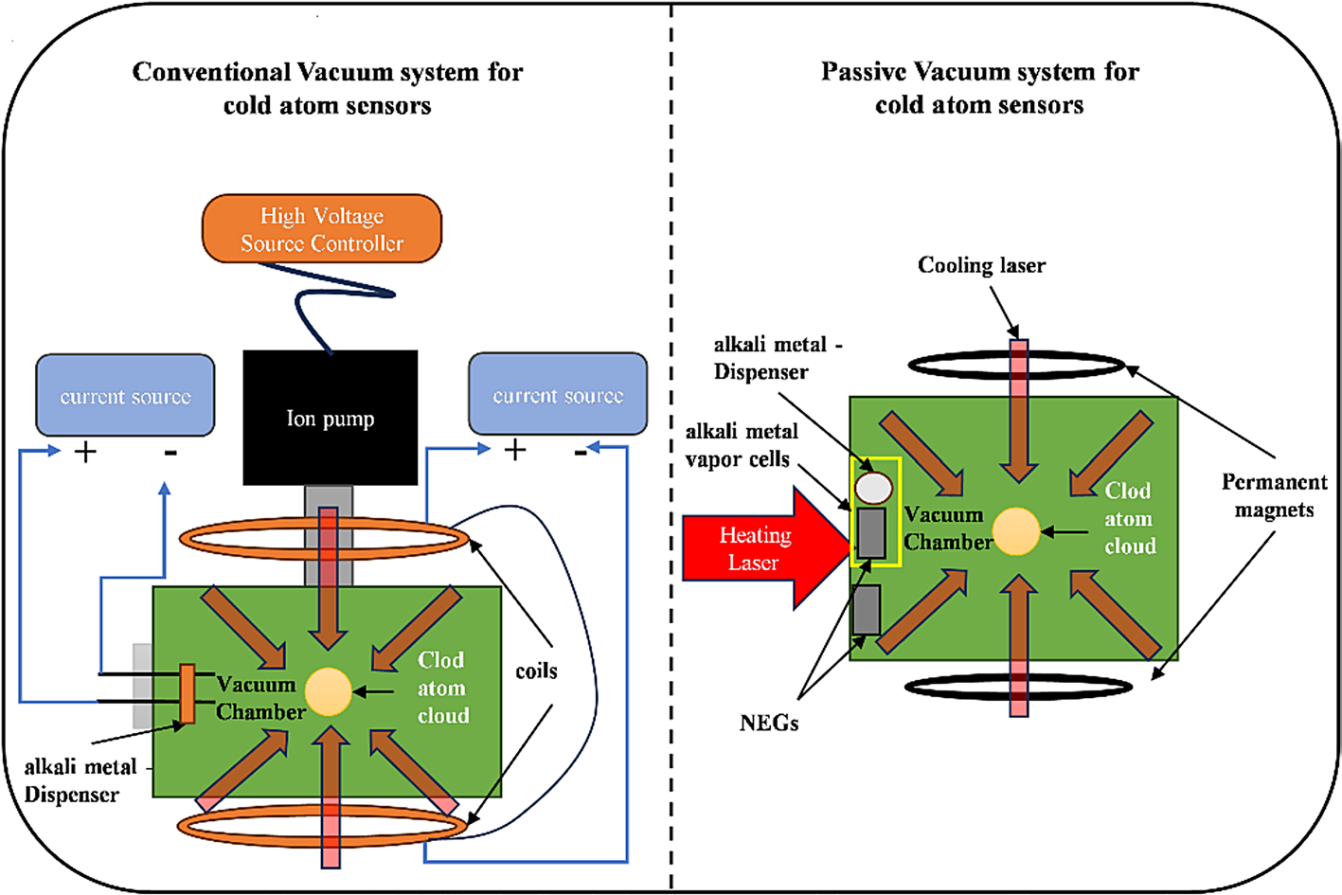
The comparison of the conventional vacuum system and PVS for cold atom sensors.

The advent of laser cooling of atoms has had an important influence on precision measurement techniques^1–3^, so that a variety of state-of-the-art quantum sensors based on the manipulation of cold atoms have been implemented successfully^4,5^. These sensors include cold-atom gravimeters^6–11^, cold atomic clocks^12–15^, which ultimately enable the transfer of such instruments from laboratory prototypes to practical applications^16–18^. This transition requires compact and light quantum sensors to realize transportable and field measurements. The system of quantum sensors can be divided into three modules: the laser module, the electronic and control module, and the VSCAS. The miniaturization of the laser module has recently been realized by the technology of integration on-chip^19,20^, and the electronic and control module is becoming compact as well. As for the VSCAS, significant progresses have been made in recent years, which offers an effective method for reducing the size, weight, and power (SWaP). As shown in Fig. 1, the VSCAS comprises a vacuum system, a beam splitting system, a magnetic field system, and an electronic system. The conventional vacuum system comprises an ion pump to ensure the maintenance of an ultra-high vacuum (UHV), a vacuum chamber, and an alkali metal source. The utilization of active vacuum components in the cold atom sensors has some problems. Firstly, it has been shown to increase power consumption. Secondly, it will increase the overall size and weight of the vacuum system. Conversely, the vacuum system based on the passive vacuum technology has a low SWaP. The PVS replaces the ion pump with getters, and consequently, it does not require an external high-voltage source. Furthermore, it replaces the feedthrough with a passive atomic source^21^, and recent research has replaced anti-Helmholtz coils with toroidal permanent magnets to generate quadrupole magnetic fields with a sufficiently high gradient to trap atoms^22,23^. All of these alternatives will consume no additional energy and will allow the cold atom sensor to be smaller in size and lighter in weight. Therefore, PVS is an effective way to reduce the SWaP of the vacuum system for cold atom sensors. It has been demonstrated in numerous studies^24–26^ that passive maintenance of miniaturized ultra-high vacuum chambers is capable of meeting experimental requirements. This represents the limit of passive vacuum maintenance verification. Another study has focused on the verification of vacuum detection in vacuum chambers by MOT loading^27–30^. This technology can replace traditional vacuum gauges or ion pumps with a high-voltage controller.

However, the vacuum level achieved by these passive vacuum designs has a common problem that getters alone cannot trap some inert gases, especially helium, and the ultimate vacuum of the chamber in the final vacuum system does not reach a high level, and the current passive pumps independent of the ion pumps are generally used to maintain the minimum air pressure in the vacuum chamber at 10^-5^ Pa^31^. Therefore, to meet the demands of more precise cold atom experiments, we should select a more suitable passive vacuum system solution and focus our research on key factors such as helium permeation through the glass chamber, outgassing rate of the metal chamber material, pumping speed of the non-evaporable Getter (NEG), and its lifetime. In the future, if we continue to miniaturize cold atom vacuum systems—even integrating MEMS processes to achieve on-chip cold atom sensors—and integrate all laser, vacuum, and electronic control modules into a miniature vacuum chamber, verifying the effectiveness of passive vacuum technology is a critical step toward realizing this goal.

In this paper, we summarize the latest progress of passive vacuum technology, which is important for the simplification of the VSCAS. Firstly, the materials of low helium-permeability are shown to give a selection for PVS. Secondly, the key components for PVS are analyzed, which include the getters, simplified atomic source, and miniaturized vacuum chambers. Thirdly, the methods of integration for PVS are discussed so that the low SWaP atomic sensor can be implemented. Besides, the evaluation of the pressure has been given so that the performance of PVS can be well estimated. Finally, we conduct a series of preliminary experiments and present the corresponding results. Moreover, the application prospects of PVS are analyzed.

## Fundamental theory of the passive vacuum system

### Gas permeation and outgassing dynamics in an ultra-high vacuum system

**Fig. 2:**
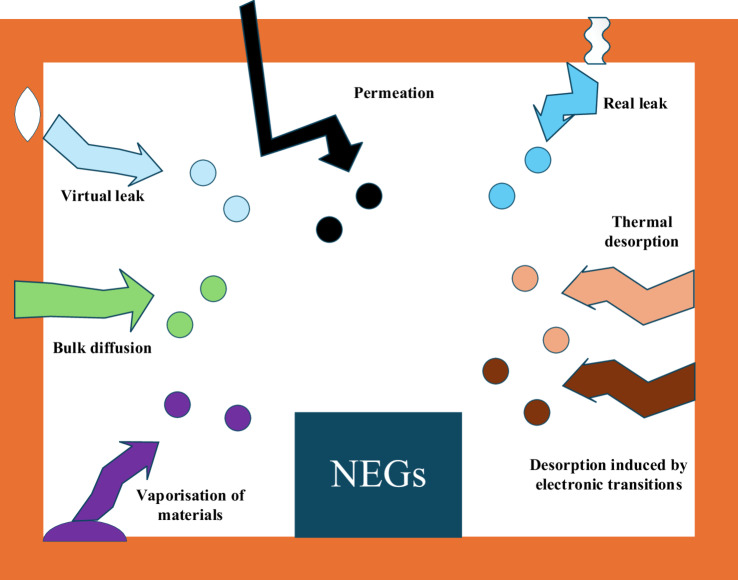
Gas sources in PVS.

The achievement of UHV for compact cold-atom systems is predicated upon the comprehensive suppression of gas permeation and material outgassing^32,33^. As shown in Fig. 2 Gas sources in PVS, the gas sources in a typical vacuum system involve multiple generation mechanisms, primarily the following: (a) System leak (Including both real and virtual leaks); (b) Thermal desorption; (c) Desorption induced by electronic transitions; (d) Vaporization of materials; (e) Gas diffusion from the bulk and subsequent desorption; (f) Gas permeation through the walls.

**Fig. 3:**
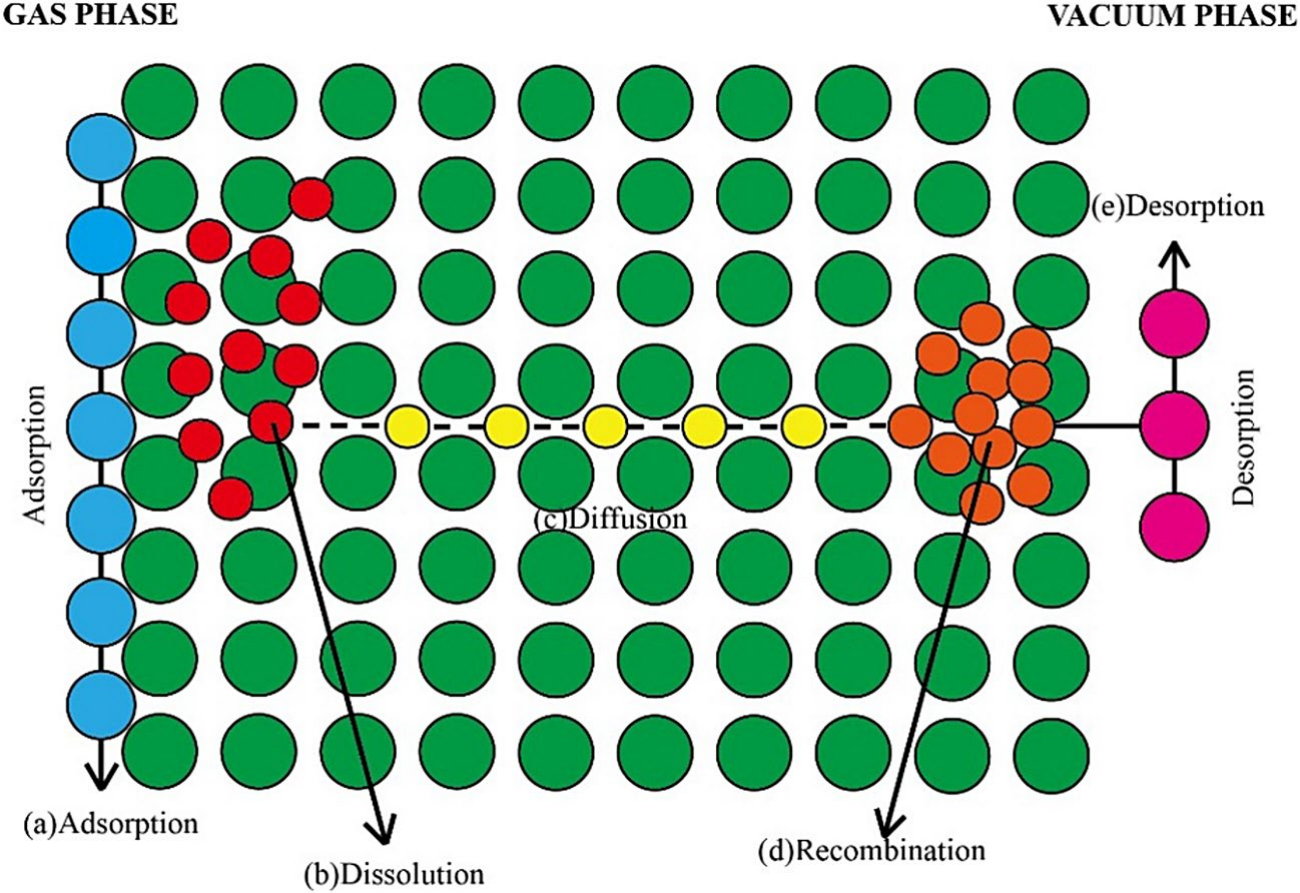
Gas permeation in vacuum systems.

NEG pumps have been shown to be highly effective at removing reactive gases (H₂, CO, N₂). However, their inability to adsorb noble gases, particularly helium with a kinetic diameter of 0.28 nm, imposes stringent requirements on the material selection for the chamber. Thus, we begin our analysis with the fundamental physical theory of gas permeation. The gas permeation process can be divided into the following steps (Fig. 3): (a) Gas dissolves into the gas chamber wall; (b) Gas dissolves into the material; (c) Gas diffuses through the gas chamber wall to the inner wall of the vacuum chamber; (d) Gas atoms recombine to form molecules; (e) Gas desorbs into the interior of the vacuum chamber. The dissolution of gases follows Henry’s Law^34,35^:

1 $$c = sP^{n}$$

where *c* is the concentration of the gas in the solid, *P* is the gas pressure, *s* is the solubility, and *n* is a material-dependent coefficient (*n* = 1 for nonmetallic materials). Diatomic molecules undergo dissociation during dissolution, so their *n* value is typically 1/2. After dissolving, the gas diffuses through the solid along the concentration gradient. At steady state, the diffusion process follows Fick’s first law of diffusion^34^. The gas diffusion flux Q per unit cross-sectional area per unit time is expressed as:

2 $$Q = - D\frac{dc}{{dx}}$$

where *D* is the diffusion coefficient, and ${{dc} \mathord{\left/ {\vphantom {{dc} {dx}}} \right. \kern-0pt} {dx}}$ is the concentration gradient. Consider a wall of thickness *d* and large surface area (neglecting edge effects). At time *t* = 0, its outer and inner surfaces are exposed to pressures *P*_*1*_ (outer, e.g., atmospheric pressure) and *P*_*2*_ (inner, e.g., vacuum), respectively. According to Henry’s law, the gas concentrations at the two surfaces are $c_{1} = sP_{1}^{n} ,c_{2} = sP_{2}^{n}$. Substituting the concentration gradient into Fick’s first law yields the steady-state gas permeation flux^36^:

3 $$Q_{permeation} = \frac{{Ds(P_{1}^{n} - P_{2}^{n} )}}{d}$$

Among these, the permeation constant is referred to *Ds = K*. In practical scenarios, the system often requires a considerable amount of time to reach a steady state. Therefore, the permeation behavior during the transition state must be described by Fick’s second law^35^:

4 $$\frac{\partial c(x,t)}{{\partial t}} = D\frac{{\partial^{2} c(x,t)}}{{\partial x^{2} }}$$

Considering the initial and boundary conditions:

5 *c(x,t = 0) = f(x)*

6 $$c(x = 0,t) = sP_{0}^{n} = c_{1}$$

and

7 $$c(x = d,t) = sP_{d}^{n} = c_{2}$$

The long-term steady-state solution obtained by substituting into the Eq. (4) is^35^:

8 $$Q(t) \approx \frac{{KP_{0}^{n} }}{d}\left( {t - \frac{{d^{2} }}{6D}} \right)$$

The time constant $t_{a} = {{d^{2} } \mathord{\left/ {\vphantom {{d^{2} } {6D}}} \right. \kern-0pt} {6D}}$ is the duration required for *Q* to be valid. This time constant is crucial for determining the *D* value. By expressing *Q* as a function of *t*, the $t_{a}$ value can be determined through extrapolation of the straight line, thereby enabling the calculation of *D* and *K* .

The dissolution, diffusion, and permeation of gases in solids all require activation energy, which is an exponential function of temperature^37^.

9 $$\begin{gathered} D = D_{0} \exp \left( {{{ - E_{D} } \mathord{\left/ {\vphantom {{ - E_{D} } {RT}}} \right. \kern-0pt} {RT}}} \right) \hfill \\ S = S_{0} \exp \left( {{{ - E_{s} } \mathord{\left/ {\vphantom {{ - E_{s} } {RT}}} \right. \kern-0pt} {RT}}} \right) \hfill \\ K = K_{0} \exp \left( {{{ - E_{K} } \mathord{\left/ {\vphantom {{ - E_{K} } {RT}}} \right. \kern-0pt} {RT}}} \right) \hfill \\ \end{gathered}$$

The temperature-dependent transport coefficients *D*_0_, *S*_0_, and *K*_0_ are intrinsically governed by gas-solid pair interactions, where *D*_0_ reflects the intrinsic diffusivity at infinite temperature, *S*_0_ is the equilibrium solubility limit, and *K*_0_ is the inherent permeability constant. These parameters, when considered in conjunction with activation energies for diffusion *E*_*D*_, dissolution enthalpy Δ*H*, and permeation *E*_*K*_, where *R*=8.31441 J/(mol⋅K) and *T* is the absolute temperature, provide a comprehensive framework for understanding the mechanisms underlying these processes.

At the same time, during manufacturing processes and atmospheric storage, solid materials have been observed to dissolve and adsorb certain gases. When placed in a vacuum environment, gas release phenomena are observed, which can be attributed to physical processes such as adsorption, dissolution, diffusion, and permeation. In an actual vacuum system, the outgassing rate of materials follows a power-law relationship^38,39^:

10 $$q = q_{0} t^{{{ - }\gamma }}$$

Where *q* denotes the outgassing rate, $q_{{0}}$ is the initial outgassing rate of the material, *γ* is the power-law exponent for outgassing rate decay, and its value typically ranges from 0.9 to 1.3. When the pumping speed of the system remains constant, the pressure variation in the system also follows a power-law form^39^.

11 $$P = kt^{ - \gamma }$$

where *P* is the system pressure, *k* is the proportional coefficient satisfying $k = {{q_{0} } \mathord{\left/ {\vphantom {{q_{0} } {S_{eff} }}} \right. \kern-0pt} {S_{eff} }}$, and $S_{eff}$ is the effective pumping speed.

**Fig. 4:**
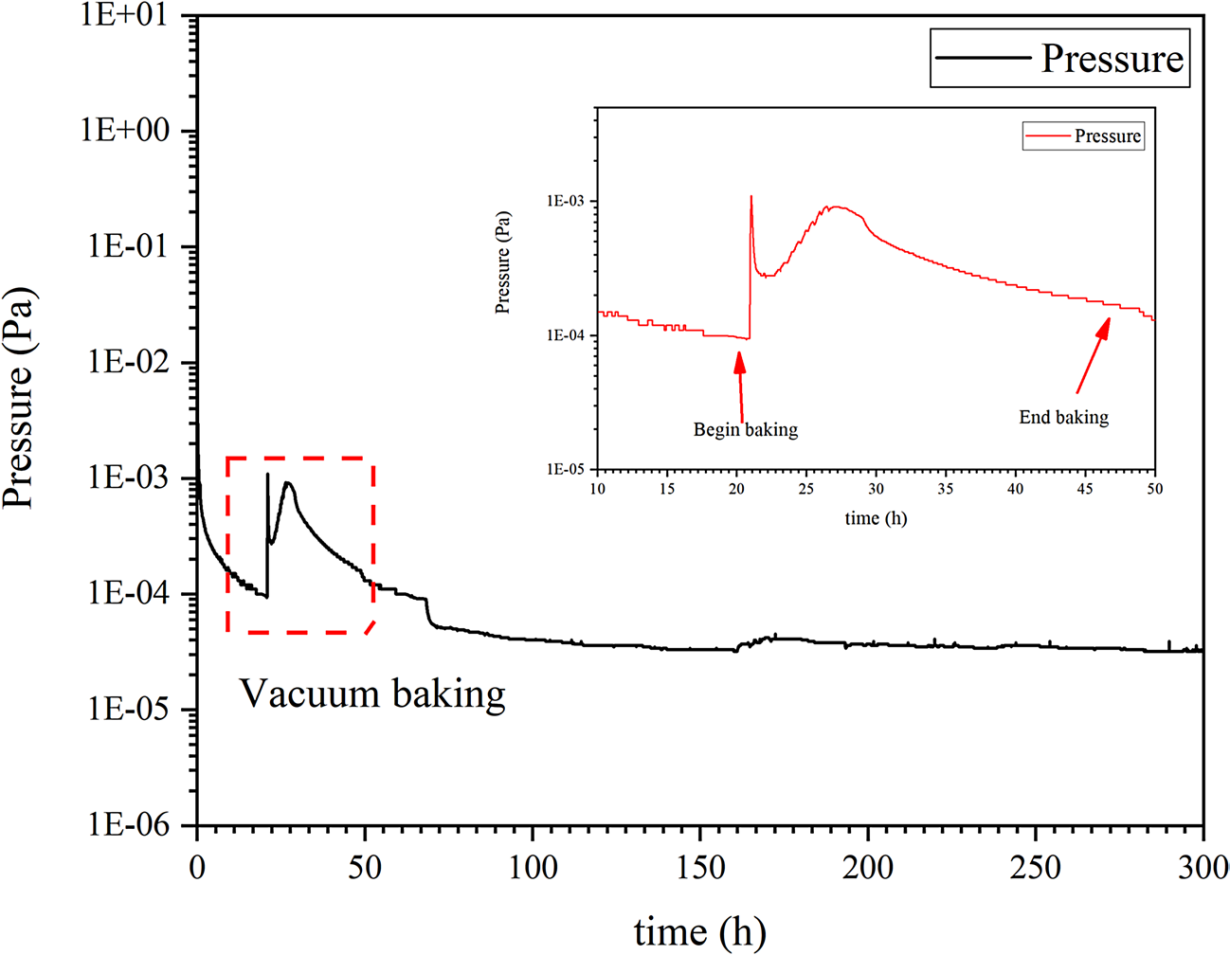
Pressure evolution of the complete vacuum preparation process.

Figure 4 shows the full recording of the vacuum preparation process for one of the quantum sensor setups in our laboratory. The variation in the material outgassing rate can be observed from this figure. At the initial startup of the turbo-molecular pump, the outgassing rate is relatively high but decreases rapidly, allowing the vacuum system to reach a high vacuum level within a few tens of minutes. Subsequently, to accelerate the vacuum preparation process, baking is applied to speed up material outgassing. The outgassing rate rises rapidly to a peak and then decreases gradually, corresponding to the baking stage during vacuum preparation. The vacuum pressure rises quickly to a peak and then declines slowly. After a period of baking, the temperature returns to its pre-baking level, while the outgassing rate becomes much lower than before baking. Therefore, baking is an effective method for ultrahigh vacuum preparation.

Subsequently, the quantity of gas emanating from the surface of the container wall per unit time is as follows:

12 $$Q_{Outgas\sin g} = A \cdot q$$

$Q_{Outgas\sin g}$ represents the amount of gas released from the surface of the container wall, and *A* is the area of the outgassing surface.

It is evident that a range of methodologies is effective in impeding the penetration of gas molecules. These methodologies include surface coating, vacuum high-temperature baking, the thin film barrier layer method, the surface vacuum metal film plating method, and high-precision detection and leakage plugging of leakage points. The outgassing rate of the material surface can be mitigated through material selection and process treatment. Subsequently, the miscellaneous gases adsorbed on the surface of the chamber can be removed by high-temperature baking.

### Dynamic maintenance of the passive vacuum system

The pumping mechanism employed in passive vacuum is known as chemisorption, and the pumping agent is referred to as a getter. During the adsorption process, an exchange of electrons occurs between the atoms of the solid and the gas molecules, resulting in a phenomenon analogous to the force of a chemical bond. Chemisorption exhibits a certain degree of selectivity, albeit exclusively between a specific solid and a particular gas. In the process of adsorption, the gas molecules must be decomposed into atoms, and a certain amount of heat energy is required; only molecules with a greater or equal amount of heat energy can be adsorbed. Subsequently, a chemosynthesis reaction occurs, resulting in the release of heat. The process of chemosynthesis requires the formation of chemical bonds, thereby restricting chemisorption to the formation of a solitary molecular adsorption layer. In the event of the presence of a molecule at the site of incidence, the possibility of the adsorption of other molecules at this site is precluded; solely those molecules incident on the adsorption vacancies on the surface can be adsorbed. However, there is also a certain risk that the adsorption will become unstable and detach from the surface after this. The ratio of the number of adsorbed molecules to the number of incident molecules is denoted as the sticking probability^35^:

13 $$\alpha_{c} = \alpha_{p} f(\theta )\exp \left( {{{ - E_{a} } \mathord{\left/ {\vphantom {{ - E_{a} } {RT}}} \right. \kern-0pt} {RT}}} \right)$$

The sticking coefficient $\alpha_{p}$ is a measure of the probability that a molecule incident on an adsorption vacancy will be adsorbed. The function *f(θ )* is contingent on the extent of surface coverage *θ*($\theta = {{N_{s} } \mathord{\left/ {\vphantom {{N_{s} } {N_{m} }}} \right. \kern-0pt} {N_{m} }}$), with *N*_*S*_ denoting the number of adsorbed molecules per unit area and *N*_*m*_ representing the total capacity of the surface to accommodate molecules. It is essential to ascertain whether this function serves as a reliable metric for the probability of efficient molecule adsorption, with the caveat that the molecules must not detach from the surface post-collision. The coverage function, denoted *f(θ )*, is associated with the adsorption space occupied by the molecule. Monoatomic gas occupies a single adsorption space, then $f(\theta ) = 1 - \theta$. Diatomic gas, on the other hand, occupies two adsorption spaces, then $f(\theta ) = {{Z(1 - \theta )^{2} } \mathord{\left/ {\vphantom {{Z(1 - \theta )^{2} } {Z - \theta }}} \right. \kern-0pt} {Z - \theta }}(1 - \theta )^{2}$. The number of adsorption positions around an adsorption position is generally taken as *Z* = 4. In instances where the coverage is minimal, then $f(\theta ) = (1 - \theta )^{2}$. Let $\alpha_{0} = \alpha_{p} \exp \left( {{{ - E_{a} } \mathord{\left/ {\vphantom {{ - E_{a} } {RT}}} \right. \kern-0pt} {RT}}} \right)$, $\alpha_{0}$ is the initial sticking coefficient, Eq (14) can be written as:

14 $$\alpha_{c} = \alpha_{0} (1 - \theta )$$

Under pressure *P* and gas temperature *T*, the number of molecules incident on a unit surface per unit time is^35^:

15 $$\phi = \frac{dn}{{dt}} = \alpha_{c} \frac{P}{{\sqrt {2\pi mKT} }} = 8.32 \times 10^{27} \alpha_{c} \frac{P}{{\sqrt {MT} }} = \lambda_{1} \alpha_{c} P$$

In the formula, *ϕ* is the gas adsorption rate (${\mathrm{(m}}^{2} \cdot {\mathrm{s)}}^{ - 1}$), *m* is the gas molecular mass, *K* is the Boltzmann constant, *P* is the gas pressure, *T* is the gas temperature, $\alpha_{c}$ is the adhesion probability, *M* is the molar mass of the gas, and $\lambda_{{1}}$ is the gas adsorption constant.

In contrast, the desorption rate constant of the gas can be written as^39^:

16 $$\lambda_{2} = \nu \exp \left( {{{ - E_{a} } \mathord{\left/ {\vphantom {{ - E_{a} } {RT}}} \right. \kern-0pt} {RT}}} \right)$$

Where *ν* is the frequency factor, 10^13^ s^-1^^35^. Therefore, at a given pressure and temperature, the adsorption and desorption of the gas reach a certain equilibrium^40^.

17 $$\phi = \lambda_{{2}} \theta$$

The amount of adsorbed gas under the final dynamic equilibrium is

18 $$\theta \left( {P\lambda_{{1}} \alpha_{{0}} { + }\lambda_{{2}} } \right) = \lambda_{{1}} \alpha_{{0}} P$$

That is:

19 $$\theta = \frac{{\lambda_{1} \alpha_{0} }}{{\lambda_{2} }}\frac{P}{{1 + \frac{{\lambda_{1} \alpha_{0} }}{{\lambda_{2} }}P}}$$

Let $A = \frac{{\lambda_{1} \alpha_{0} }}{{\lambda_{2} }}$, then the equation can be written as:

20 $$\theta = \frac{AP}{{1 + AP}}$$

## Materials of the passive vacuum system

The development of UHV chambers for cold atom systems is motivated by the need to address vacuum lifetime constraints for deployable cold-atom sensors. Miniaturization requires compact geometries, reduced weight, and passive pumping systems that balance chamber size with optimized gas conductance. Passive vacuum maintenance imposes stringent requirements on material selection, where chamber components must exhibit low gas permeability (particularly for helium), minimal outgassing, and mechanical stability while ensuring compatibility with precision machining processes. It is imperative to note that critical material and component categories include the main chamber, optical viewports, and sealing interfaces. It is important to emphasize that each of these categories demands tailored solutions to ensure long-term vacuum integrity. This integrated approach prioritizes material performance and system design to advance the development of portable cold-atom systems.

### Metal material

Metallic materials are processed by melting and casting, and the molecular hydrogen, oxygen, nitrogen, and carbon oxides in the air are dissolved in the materials. Based on the storage of material, a number of gases are adsorbed by the surface of metal, such as water vapor, oxygen, nitrogen, and carbon oxides. The re-contamination of the material during processing, in conjunction with the penetration caused by its own non-dense nature, is the primary source of gases in a vacuum. The metals employed in vacuum systems principally comprise iron, copper, aluminum, and titanium-based metals and alloys. The metals most frequently utilized in laboratory settings are stainless steel, aluminum, copper, and titanium, which exhibit very low intrinsic outgassing rates^34^ (Table 1). In the context of the prevailing cold atomic vacuum system, cavity, piping, flanges, and a range of vacuum components, stainless steel and titanium are the most commonly employed materials. Stainless steel, utilized as the primary material, exhibits minimal permeability to helium. However, in comparison to titanium, stainless steel has a higher outgassing rate of hydrogen retained inside the material^41^. In the case of stainless steel, outgassing is due primarily to the hydrogen trapped in the bulk steel when it is produced, not from permeation due to hydrogen outside the vacuum chamber. Consequently, titanium is currently employed in the construction of ultrahigh vacuum chambers, a practice that ensures the maintenance of the ultrahigh vacuum function in this domain. Such machined metal cavities are also used in miniaturized vacuum chambers^15,42^. Furthermore, gold, silver, copper, and aluminum are conventionally employed as sealing materials, while fusible alloys are utilized as seals for glass, metal, and ceramics

**Table 1:** Outgassing rate of commonly used metallic materials in vacuum systems.

Material	*Q* (Pa⋅m^3^/s⋅m^2^)	Test time (h)	Test temp. (°C)	Method of handling	Ref
316L stainless steel	4.0 × 10^−8^	100	20	Untreated	^43^
304 stainless steel	1.46 × 10^−13^	70	20	Bake at 250°C for 330 hours	^44^
316L stainless steel	1.59 × 10^−13^	70	20	Bake at 900°C for 4 hours and electrolytically polish	^44^
6061T-6 aluminum	1.46 × 10^−13^	70	20	Chemical cleaning and bake at 250°C for 30 hours	^44^
TA6V titanium	< 2.6 × 10^−10^	24	25	Bake at 140°C for 24 hours	^45^
Oxygen-free high-conductivity (OFHC) copper	5.4 × 10^−12^	168	20	Bake at 300°C for 48 hours and electrolytically polish	^46^

### Transparent non-metallic materials

Despite the inherent restrictions on scalability due to miniaturization of the cavity, it is imperative that the vacuum chamber incorporate optical access. This optical access is imperative for the facilitation of laser cooling processes and the precise measurement of atomic signals. However, it is vital that the introduction of optical access does not cause any significant reduction in the vacuum performance of the chamber.

Ideally, the vacuum viewing window would satisfy multiple criteria concurrently. Firstly, it is imperative that the material remain impermeable to inert gases. Secondly, it is imperative to ascertain the viability of an adhesive method that is conducive to large-scale production for its attachment to the chamber body. Thirdly, the apparatus should display appropriate transmissivity at atomic wavelengths. Furthermore, the vacuum viewing window should be free from birefringence. The Table 2 illustrates the range of low-permeability window materials that are currently available.

**Table 2:** Low helium-permeability optical materials.

Material	*K*_*He*_ (cm^2^/s)	Test Temp. (°C)	Ref
Quartz (SiO_2_)	1.14 × 10^−10^	25	^37^
Ceramic (Al₂O₃)	10^−14^		^36,37^
Pyrex	1.5 × 10^−11^	25	^36^
Ceramic (ZrO₂)	10^-14^-10^−13^		^47^
Borosilicate Glass	9.2 × 10^−13^	25	^36,48^
Phosphate	1.9 × 10^−14^	28	^36^
Soda-lime glass	7.5 × 10^−15^	25	^36^
X-ray shield glass	1.4 × 10^−15^	84	^36^
Lead glass with boric acid	1.9 × 10^−15^	158	^36^
Aluminosilicate Glass	1.4 × 10^−12^	91	^48^
Sapphire	2.0 × 10^−21^	70	^49^

## Components of the passive vacuum system

### Vacuum chamber

In the quantum domain, ultra-high vacuum chambers must not only account for material properties such as outgassing and permeation but also reduce weight and volume to facilitate miniaturization of next-generation sensors. Furthermore, they must satisfy experimental requirements for optical interactions and detection in quantum measurements, as well as reaction characteristics with alkali metal sources. Currently, vacuum cavities in cold atom sensors are predominantly fabricated from metals like 304, 316L stainless steel, or titanium. In order to reduce the weight and volume of vacuum systems while increasing the optical aperture, all-glass vacuum cavities have seen rapid development in recent years. However, some of these glass cavities remain constrained by integration processes and inevitably require metal cavities as transition chambers or connectors.

In addition to metals, a favorable material for fabricating cold-atom vacuum chambers has been identified in recent studies as low-temperature co-fired ceramics (LTCC), which are commonly used for vacuum sealing^50^ and as components of optical cavities. LTCCs are non-reactive to alkali metals and can withstand ultrahigh vacuums. LTCC-based chambers^51^ facilitate the customization of cubic geometries with integrated optical ports; however, their fabrication requires precise high-temperature sealing processes. Despite the limitations in scalability when compared with silicon-based methods, LTCC provides a promising platform for bespoke vacuum assemblies.

**Fig. 5:**
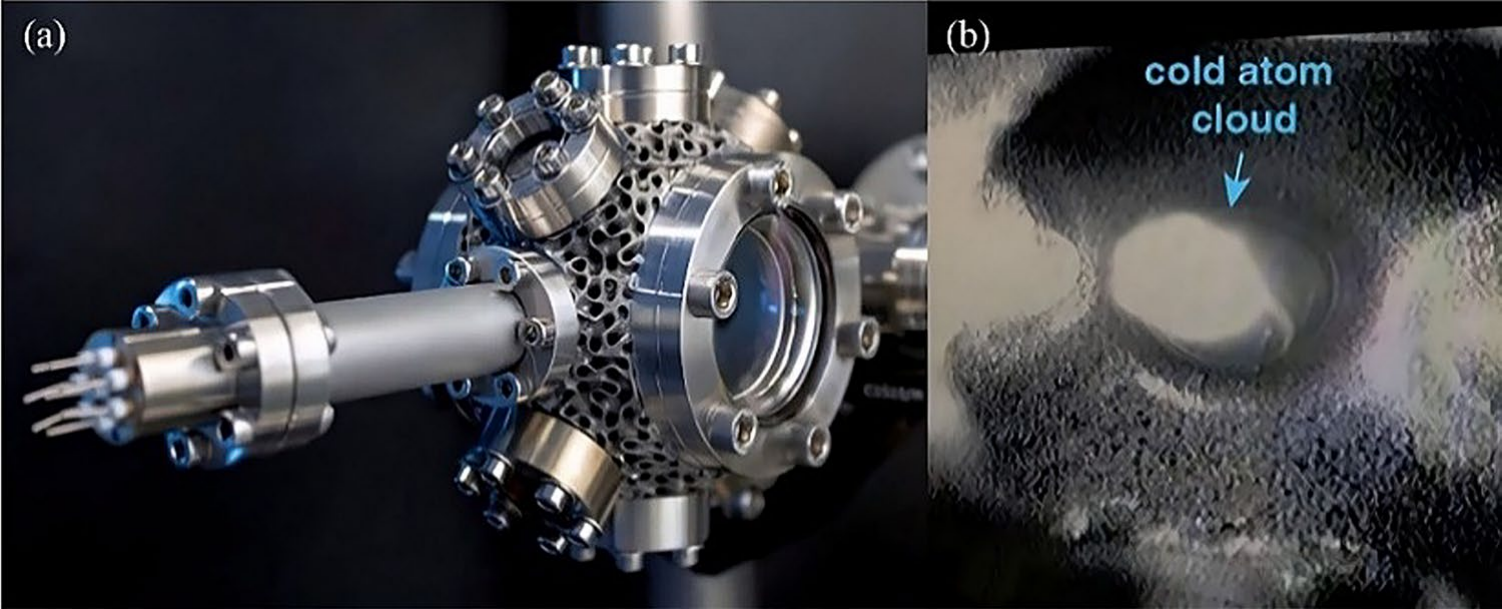
(**a**) The image depicts a three-dimensional printed octahedral vacuum chamber, predominantly composed of aluminum alloy (AlSi10Mg). During the printing process, an oxide layer (Al₂O₃, thickness ≈ 20 nm) is formed on the surface of the chamber, thereby inhibiting gas permeation (mainly helium). (**b**) Cold atom experiments conducted within the 3D-printed vacuum chamber have successfully observed cold atom clouds.

Additive manufacturing has further revolutionized the fabrication of UHV chambers^52^, enabling the production of 3D-printed alloy chambers with significantly reduced weight and size (Fig. 5a). The mass of this chamber is less than one-third of that of a conventional stainless-steel chamber, with a weight of merely 245 g. Through comprehensive analyses using mass spectrometry, scanning electron microscopy (SEM), and X-ray photoelectron spectroscopy (XPS), it has been revealed that the surface oxide layer generated during the 3D printing process plays a pivotal role in enhancing the vacuum performance.

The UHV chamber has been demonstrated to function within a pressure range of 10^−7^ Pa for a duration of 48 hours under passive pumping conditions. Furthermore, upon reactivation of the ion pump, it has been demonstrated that the pressure can be restored to below 10^−8^ Pa within a period of 6 minutes.

With regard to the practical applications of the UHV chamber, its employment has resulted in the successful capture of a cold ^85^Rb atomic cloud (Fig. 5b). This accomplishment not only validates its applicability in quantum technologies but also exemplifies the significant breakthrough of 3D printing technology in the fabrication of UHV chambers. Consequently, this finding paves the way for novel possibilities in the future development of quantum technologies and precision timing devices.

### Getter pump

This objective aims to develop a UHV system without an active pumping mechanism. Such systems can reduce overall size, lower power consumption requirements, and decrease system costs. Passive pumping sources consist of getter materials—metals or alloys that chemically adsorb active gases (O₂, CO, N₂, and H₂) in high-vacuum environments. Getter materials are typically categorized into two types: evaporable getters and non-evaporable getters. Evaporable getters are generally metals deposited onto the walls of vacuum system chambers by heating to increase vapor pressure, with typical materials being barium^53^ and titanium. This process requires heating to very high temperatures to induce displacement reactions in metal compounds, forming elemental metals. Reaction temperatures generally exceed 800°C. NEG absorbers also chemically adsorb gases on their surfaces. When heated at high temperatures, the oxide layer on the metal surface can diffuse into the metal body, exposing a clean and highly reactive surface. The activation temperature range for NEG is approximately 200-800°C. However, evaporable absorbers are generally suited for large-volume, high-surface-area vacuum systems. Their relatively high activation temperature may cause evaporated metal to migrate to other system components. Therefore, selecting NEG for miniature cold-atom PVS is a reasonable choice.

NEGs are mainly composed of group IV/V metals and their alloys, including titanium (Ti), zirconium (Zr), vanadium (V), hafnium (Hf), etc. These alloys may also contain metals such as aluminum (Al) and iron (Fe). In a vacuum environment, these elements exhibit high oxygen solubility, high diffusion coefficient, and high adsorption enthalpy for various gases^54^. Currently, NEG is commonly used as a trap vacuum pump in the form of discs^55,56^, plates^57^, or thin films^58^. When exposed to air, NEG undergoes rapid surface passivation, forming a protective layer composed of oxides, nitrides, and carbides^59^. It requires heating and reactivation in a vacuum environment to function. The standard oxygen solubility of NEG compounds is approximately 10%, meaning a 1 μm film can undergo about 100 reactivation cycles after exposure to air. However, it should be noted that pumping efficiency begins to decline after several cycles^60^, corresponding to a total capacity of approximately 10^12^ molecules per cubic centimeter. However, the adsorption capacity of NEG components is subject to a limiting factor: when the hydrogen cycle load concentration exceeds the recommended value, the material may experience spalling or hydrogen embrittlement^61^. Furthermore, when deposited as thin films, NEGs act as an outgassing barrier^62^, thus converting the gas source into a pump and significantly reducing the ultimate pressure. At ambient temperature, NEGs are unable to pump out inert gases and certain hydrocarbon gases including methane. Gases such as N₂, CO, and CO₂ only undergo chemical adsorption on the surface of NEGs; the gas molecules form extremely strong chemical bonds with the getters, and the gases will not be released even at extremely high temperatures. High temperatures can promote the diffusion of gases into the bulk of the getters, and the pumping effect becomes negligible after the formation of a monomolecular layer, which is an irreversible adsorption process. However, hydrogen gas diffuses into the bulk of the getters to form a solid solution. High temperatures can cause the desorption of H₂, which is a reversible adsorption process, and the initial gas-gettering efficiency can be restored after activation and regeneration. In addition, both H₂O and hydrocarbon gases follow a mixed adsorption mechanism. After H₂O dissociates on the getter surface, hydrogen is adsorbed in a reversible manner, while carbon and oxygen are adsorbed irreversibly. For hydrocarbon gases, they need to undergo thermal cracking at high temperatures to complete the adsorption process. Research confirms that hydrogen adsorption and desorption in NEGs occur at thermodynamic equilibrium. Once the adsorption capacity reaches thermodynamic equilibrium, the relationship between hydrogen concentration, hydrogen pressure, and adsorbent temperature follows Sievers’ Law:

21 $$\log P = A + 2\log q - \frac{B}{T}$$

where *q* is the hydrogen concentration (torr⋅L/g); *P* is the hydrogen pressure (torr); *T* is the adsorbent temperature (K); *A* and *B* are material-specific constants for the adsorbent (determinable experimentally). As the alloy temperature rises, the equilibrium pressure of hydrogen increases accordingly. At this point, hydrogen can be evacuated using conventional fore pumps. This process is termed “adsorbent regeneration.” After regeneration, the adsorbent restores its original hydrogen adsorption properties. The regeneration time for specific adsorbent structures can be calculated using the following formula^57^:

22 $$t = \frac{M}{F}\left( {\frac{1}{{q_{f} }} - \frac{1}{{q_{i} }}} \right)10^{{ - \left( {A - {B \mathord{\left/ {\vphantom {B T}} \right. \kern-0pt} T}} \right)}}$$

Where *t* is the regeneration time,* M* is the mass of the absorbent, *F* is the pumping speed of the fore pump, *q*_*f*_ and *q*_*i*_ are the final and initial concentrations of hydrogen in the absorbent.

The miniature PVS of cold atom must be sealed under a UHV environment to ensure that the vast majority of gases—particularly noble gases—have been thoroughly evacuated prior to encapsulation. Furthermore, reactivation of getter materials will lead to gas release, permeation, and potential leakage. Therefore, NEG alloys, which combine extremely low activation temperatures with high pumping speeds and capacities, represent the optimal choice for PVS. In addition to the most classic and longest-used Zr-Al alloys, numerous novel NEG alloys have emerged in the field of vacuum technology over the past four decades. Table 3 provides examples of several purpose-designed representative alloys.

**Table 3:** List of typical NEG categories.

Model	Alloy materials	Activation temperature(℃)	Ref
St101	Zr-Al	750	^63–65^
St707	Zr-V-Fe	400~450	^66,67^
St171	Zr	900~1000	^45,68^
ZAO	Ti-Zr-V	120~180	^69–71^
St172	Zr-Zr-V-Fe	500	^72^
ZAO	Zr-V-Ti-Al	450~650	^73^
	Zr-V-Hf	200	^69^

Including the cryogenic-activated NEG developed by CERN, which can be activated during chamber baking (approximately 250°C) and covers all internal surfaces^74^. Research has found that titanium-zirconium-vanadium alloys with near-isotropic sputtering can be activated at 180°C. This coating has achieved a minimum vacuum level of 10^−12^ Pa at room temperature^70^. The pumping rate of NEG thin films is contingent on their surface area; consequently, the CERN team has also studied the effects of substrate and deposition parameters on increasing the pumping rate and capacity^75^. The pumping speed of NEG films depends on their surface area. Researchers have discovered that substrate and deposition parameters exert a certain influence on enhancing pumping speed and capacity^60^.

Recent studies have shown that for a new type of reactive getter^76,77^, alkali metal and alkaline earth metal atoms can react strongly with and combine with common residual gases in ultra-high vacuum environments. This implies that the application of alkali metal sources in quantum sensors can improve the degree of vacuum during operation. Experimental results indicate that the pumping speed of lithium getter films for carbon monoxide is comparable to that of Ti-Zr-V getters; however, the capacity of the former is more than 10^4^ times higher. This method has significant advantages in removing substances released during the bonding process. Due to lithium’s low vapor pressure and its ability to form stable compounds with various gases, research on relevant active getters has mainly focused on lithium.

### Alkali metal atomic source

**Fig. 6:**
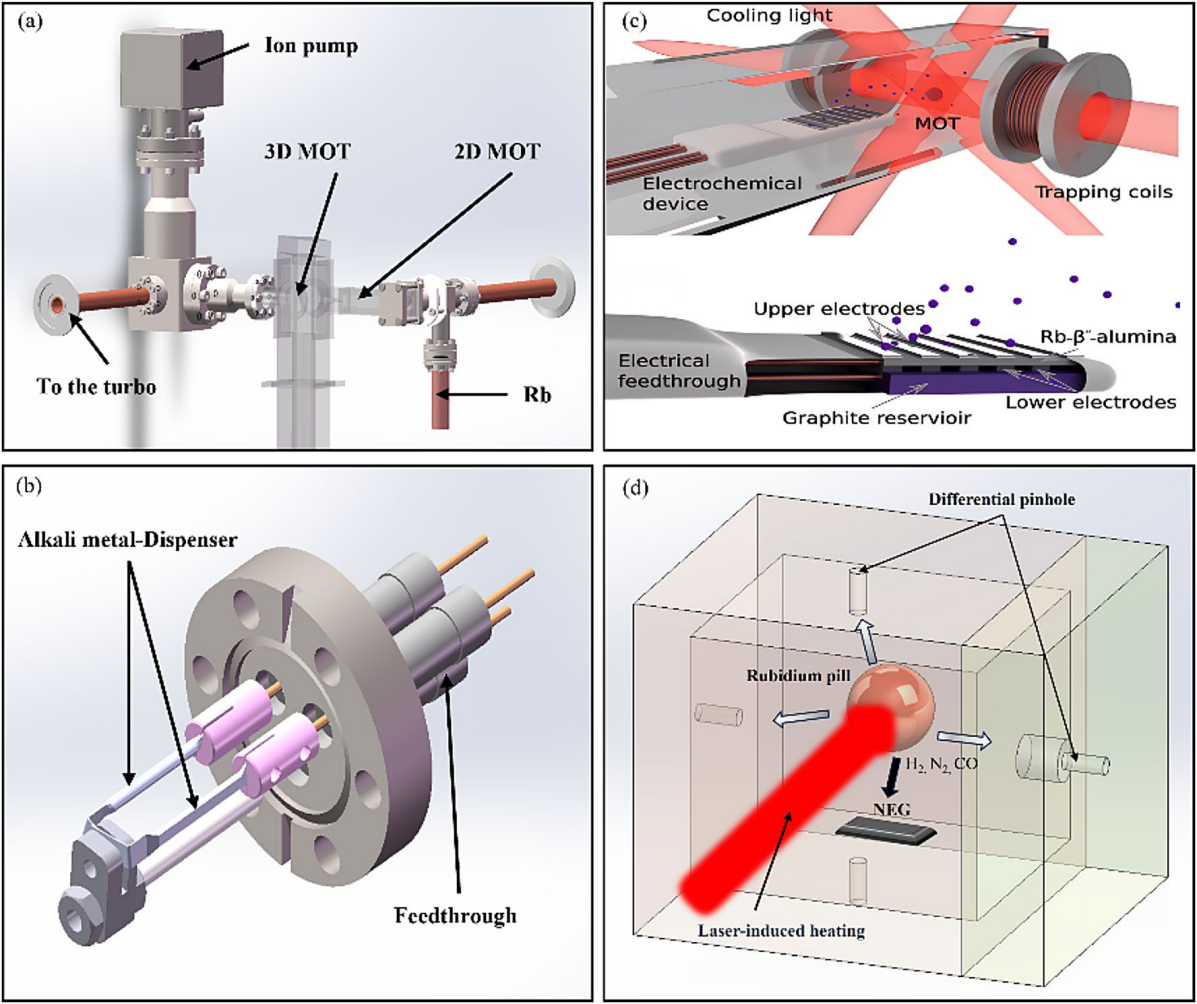
(**a**) Two-cavity differential rubidium release structure. (**b**) Commercialized AMDs. (**c**) The novel Atomic Ion Battery (AIB) is used for cold atom experiments, Reprinted figure with permission from^84^, Copyright © 2019 American Physical Society. (**d**) The Jet Rubidium Shot Program.

Atomic cooling and trapping typically require a warm atomic source to provide a suitable atomic density for forming an MOT. Loading from background vapors is a common method enabling relatively fast loading rates. However, in any quantum sensor vacuum system, precise control over the release rate of the alkali metal source is critical. This ensures uniform MOT loading, thereby preventing the occurrence of permanent vapor saturation. When thermal vapor release rates from alkali metal sources exhibit uncertainties due to material or heating inhomogeneities, supplementary control mechanisms must be implemented. Commercial alkali metal dispensers (AMDs) (Fig. 6b) are commonly used stable alkali metal compounds in quantum sensor vacuum systems^78–80^. They are composed of chromate and zirconium-aluminum getter materials. When heated, they release pure rubidium and absorb residual gases to improve vacuum quality^81^^76,77^. AMDs can be activated through Joule heating or focused laser technology^82,83^, with the latter requiring no electrical connections while reducing heat transfer. However, AMDs exhibit certain limitations in stability and response speed. They require resistive heating to release atoms during operation, a process that consumes several watts of power and is irreversible. Recent research has proposed a novel alkali metal source based on solid-state alkali ion batteries (AIBs) (Fig. 6c)^84^. This device regulates the density of alkali metal atoms within a vacuum system through electrochemical reactions, enabling precise control of atomic numbers in laser-cooled atom experiments. The battery combines low power consumption, reversibility, rapid response, and the ability to retain storage capacity after atmospheric exposure, demonstrating broad application prospects in precision measurement equipment. However, it still falls short of the theoretical requirement for absolutely zero power consumption in PVS.

The MOT loading process requires vapor pressures exceeding UHV levels, which in turn exacerbates collision and decoherence effects during subsequent operations^27^. For precision measurements susceptible to decoherence, this pressure must be reduced by another order of magnitude while precisely controlling the inflow of rubidium atoms into the MOT chamber. For such scenarios, researchers often employ multi-chamber systems combining a two-dimensional magneto-optical trap (2D-MOT) with a three-dimensional magneto-optical trap (3D-MOT) (Fig. 6a). The 2D-MOT chamber maintains a higher vapor pressure, enabling the loading of a large number of atoms into a low-velocity atomic beam. This beam is then transported through a narrow channel into the high-vacuum 3D-MOT chamber^85^. This technique has been extensively applied in the miniature cold atom systems independently developed by our research group^10,86,87^.

On the other hand, the development of chip-scale atomic clocks (CSACs) has provided multiple solutions for integrating alkali metals into PVS on-chip. These approaches include the use of pure metals^50,88^, alkali metal compounds^88^, wax particles^89^, alkali metal azides ^90^, and alkali-rich glasses^91^. However, most of these methods are unsuitable for ultra-high vacuum environments or suffer from reduced control precision and limited operational lifespan. Furthermore, pure alkali metals require high-temperature baking and bonding, and their high vapor pressure readily compromises vacuum integrity. Unless sealed during manufacturing, they are not suitable for direct use. Therefore, miniature vacuum systems necessitate research into precise release control methods. These techniques also encompass the adsorption capacity of glass and metal surfaces for alkali metals^26,27,92^and light-induced atomic desorption (LIAD) for rapid vapor pressure regulation^93–95^. However, in chip-scale systems, limited surface area restricts LIAD effectiveness. Performance can be enhanced by introducing materials with high specific surface area (provided they are fully degassed)^96,97^.

PVS for alkali metal release must simultaneously satisfy the requirements of “rapid, reversible, power-free, and miniaturizable,” characteristics crucial for developing portable cold atom physics components. Integrating existing controllable alkali metal source technologies, a preliminary passive scheme for a novel compact alkali metal source release device has recently been conceptualized^83^. This approach builds upon the previously described dual-chamber differential scheme and the bonded rubidium pellet vapor chamber^98^. The rubidium pellet is housed within a compact transparent glass chamber (Fig. 6d). This open-sided glass cavity, measuring 2 mm in diameter and 5 mm in length, can be coated internally with metallic films to facilitate alkali metal vapor recovery. The release process is controlled via LIAD technology. Ultimately, the rubidium pellet will be integrated into a miniature glass chamber within the cold atom vacuum system. An external high-energy laser beam will activate the rubidium pellet, rapidly heating it to operating temperature to release rubidium vapor within the chamber. The chamber will then rapidly fill with alkali metal vapor. The pressure differential between the vapor chamber and working chamber is controlled by adjusting the micro-pore size, thereby maintaining the ultra-high vacuum performance of the MOT vacuum chamber. It is particularly important to note that this activation method carries a risk of contaminating the vacuum chamber—the laser heating process generates accompanying gases such as H₂, N₂, and CO. The solution lies in introducing a controlled amount of NEG adsorption into the vapor chamber with micro-pores to adsorb the active gases released during alkali metal heating.

## Integration of passive vacuum system

### Method of preparation of PVS for cold atoms

The PVS for cold atoms is composed of several components, including the vacuum chamber, the vacuum flange, the vacuum observation window, the atom source, the suction agent, and others. Prior to the commencement of the preparation process, it is imperative to meticulously assess the performance index of each component. The vacuum cavity must be meticulously polished, and all components must be subjected to a rigorous cleaning process. Metal components require a minimum of three cleaning cycles, utilizing solutions such as alkali metal cleaning solution, alcohol, and deionized water, among others. It is imperative that the components be pre-degassed in a high-vacuum atmosphere baking oven, with a general temperature setting of approximately 200°C. After baking, the cleaned and degassed components are hermetically sealed and filled with an appropriate amount of nitrogen or other suitable inert gases to mitigate prolonged leakage and material outgassing. The subsequent stage of the process is the assembly of each component. Upon completion of this step, the helium mass spectrometer leak detector must be connected in order to conduct a leakage check. It is important to note that the ultra-high vacuum system has very stringent leakage rate requirements, with the system leakage rate being controlled to be less than1 × 10^−12^ Pa⋅m^3^⋅s^−1^. Following the verification of system integrity to ensure the absence of leakage, the system is connected to a turbo-molecular pump, which functions as a pre-pump, thereby establishing the requisite working pressure for the ion pump, typically in the range of 10^−5^ Pa. It is important to note that this process is often a protracted one, necessitating the application of heat to the system in order to facilitate the removal of gas and thereby accelerate material outgassing.

The activation of the getter and the atomic source is achieved by a high-vacuum preparation process. This process is accompanied by material outgassing, the release of contaminated gases through the turbo-molecular pump extraction, and the completion of a series of device activations to turn on the ion pump for ultrahigh-vacuum preparation. The vacuum pressure at this time can reach 10^−6^–10^−10^ Pa. Ultimately, the oxygen-free copper tube is severed to disconnect the ion pump from the system, leaving only the NEG to maintain the vacuum. This section summarizes the integration and preparation procedures of the PVS for cold-atom experiments.

### Compact passive vacuum system for cold atoms

**Fig. 7:**
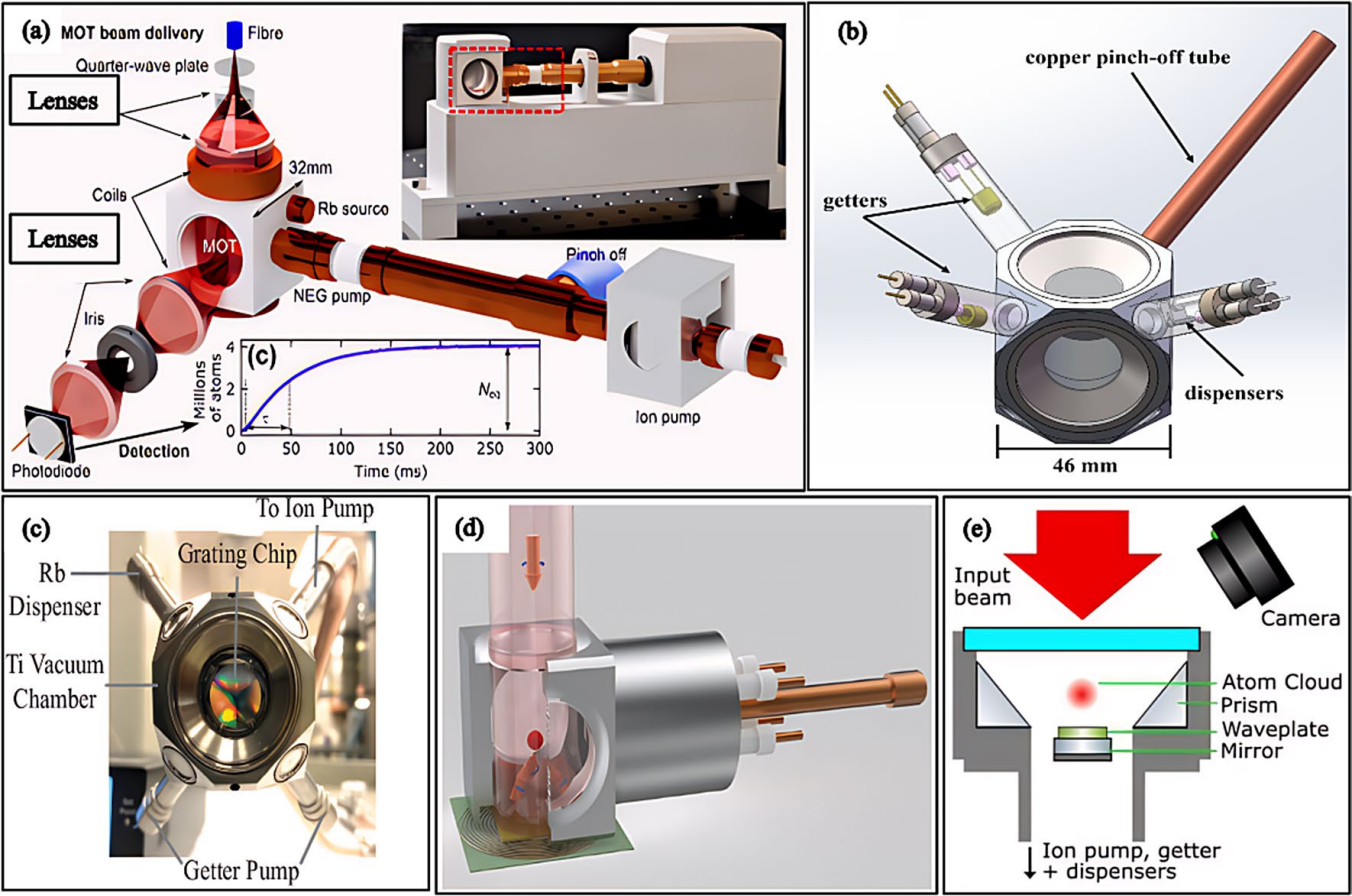
(**a**) Small ceramic sapphire passive vacuum chamber.

Currently, compact PVS based on passive vacuum technology is predominantly employed in Grating Magneto-Optic Trap (GMOT) cold atom sensors^12,99–104^. The implementation of miniaturized passive vacuum technology has significantly enhanced the practicality and stability of GMOTs. As shown in the Table. 4. This represents the current research progress in passive vacuum technology. In recent work, the Lee team pioneered the use of microfabricated gratings (Fig. 7c) and a single seed laser to construct a cold atom interferometer^105^.

**Table 4:** Comparative study of compact PVS.

Institution	Vacuum system volume (ml)/weight (g)	Passive maintenance of the ultimate vacuum pressure (Pa)	Passive maintenance time (Days)	Ref
University of Strathclyde	320 ml/ 252 g	3×10^-4^	17	^51^
University of Oklahoma	70 mL	2×10^-5^	200	^80^
Chinese Academy of Sciences	63 mL	5×10^-6^	62	^107^

This work demonstrates a condensed-matter atom interferometer capable of high-speed data transmission, laying a robust technical foundation for integrating cold atom inertial sensors into dynamic environments. This breakthrough marks a significant step toward achieving exceptional miniaturization and performance enhancement in cold atom interferometry. The team also independently reported on a PVS in a recent study, successfully operating a vacuum chamber for over 200 days using only NEGs and dispensers (Fig. 7b), with no observed vacuum degradation^80^.

The vacuum chamber is fabricated from titanium, featuring optical windows made of sapphire material with low helium permeability. C-cut windows were employed to mitigate birefringence effects while enhancing structural mechanical strength. During preparation, the system clearly integrated an ion pump, with the working chamber connected to the ion pump via oxygen-free copper tubing. Once the target ultimate vacuum of the working chamber was achieved, the oxygen-free copper tubing was sealed off to isolate the ion pump, allowing the NEG to maintain the chamber pressure passively and autonomously.

Another study measured a pressure value of 10^−5^ Pa during active pumping; under passive pumping conditions, the pressure remained stable at 3 × 10^−4^ Pa for 17 days (Fig. 7a). The team noted that another vacuum chamber was demonstrated to maintain stable pressure for over 500 days without requiring active pumping or atomic source replenishment^51^. Furthermore, a planar component integration scheme^107,109^ (e.g., combining a grating chip with a planar coil chip) reduced the vacuum chamber size to 3 × 3 × 7 cm^3^ (Fig. 7d). This vacuum system demonstrated that pressure fluctuated only slightly around 10^−6^ Pa for over 62 days under passive pumping alone. Additionally, recent deployment tests at the University of Birmingham in dynamic environments have validated the environmental adaptability of miniaturized systems. This vacuum system, reduced in size and mounted on a drone, maintains vacuum conditions during flight using a passive NEG pump (Fig. 7e). The system successfully hovered for 10 minutes and generated a cold atom cloud^108^.

**Fig. 8:**
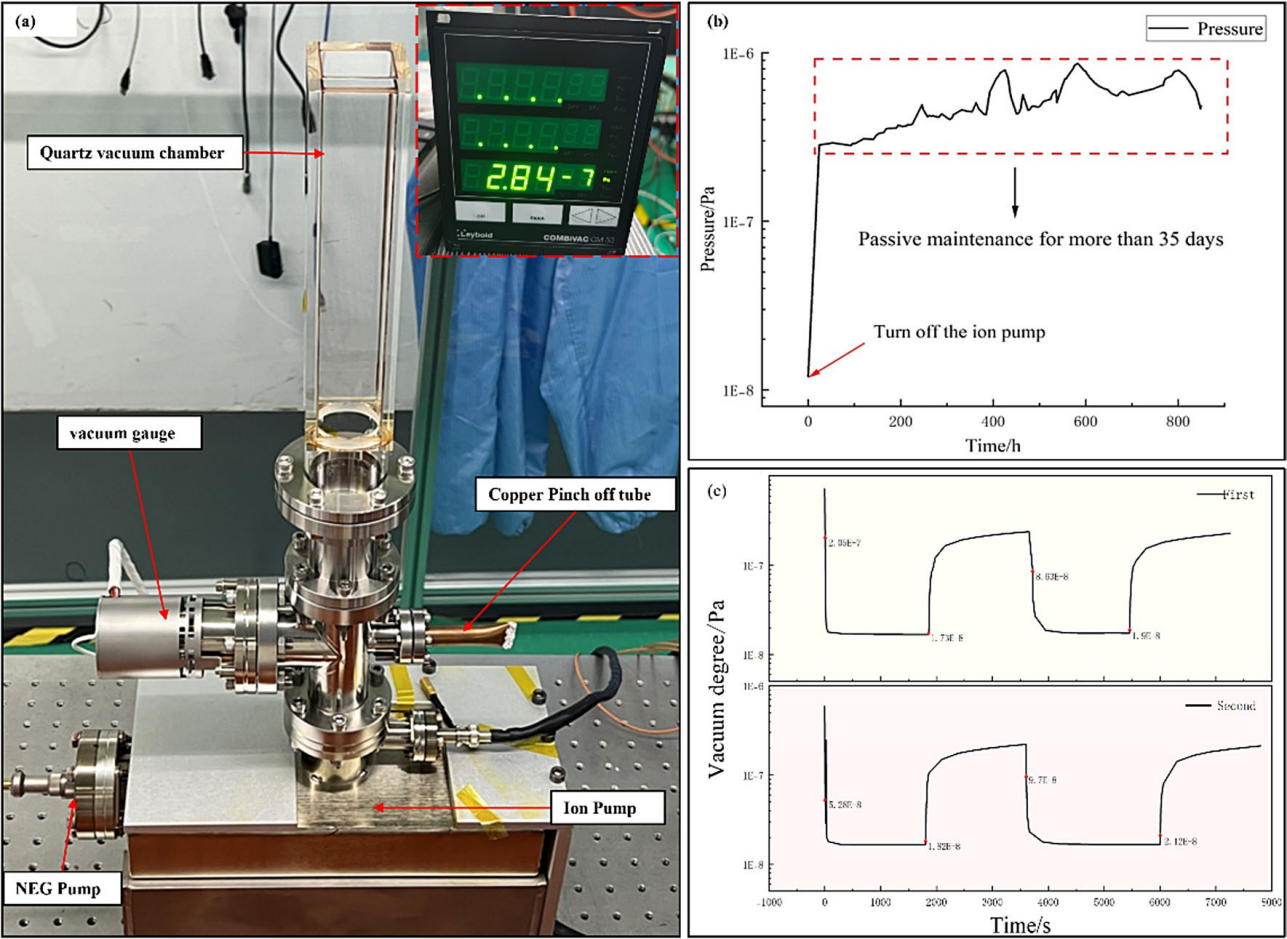
(**a**) Passive vacuum test system. (**b**) The vacuum maintenance status of the passive vacuum test system. (**c**) Experimental process of active and passive vacuum maintenance.

**Fig. 9:**
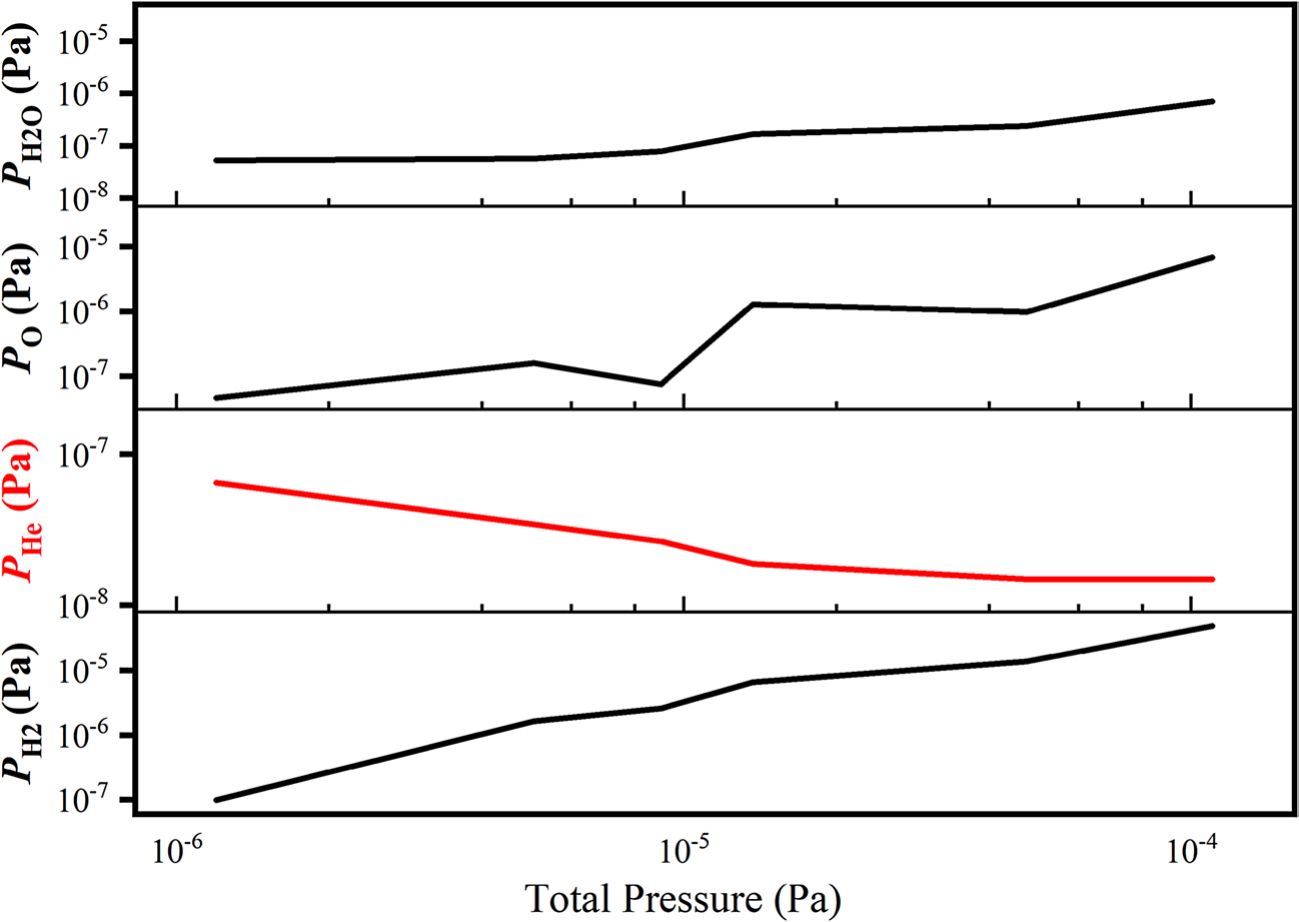
Helium partial pressure monitoring. Under ultra-high vacuum conditions, the partial pressures of other gases decrease continuously with pumping by the vacuum system, while the helium partial pressure shows an increasing trend instead.

We establish a test platform (Fig. 8a) in this study to verify the long-term stability of the passive vacuum system and determine the ultimate pressure that a standalone NEG pump can maintain in UHV conditions. The platform comprises a compound pump (75 L/s ion pump and 200 L/s NEG pump), a quartz glass vacuum chamber measuring 48 × 48 × 188 mm^3^, an ionization vacuum gauge, and a turbo-molecular pump. We connect all components using 35 mm inner-diameter stainless-steel four-way tubes with unpolished inner surfaces to simulate practical industrial manufacturing conditions. We outline the experimental procedure as follows:

Initial pumping stage: After baking, we bake the system and then evacuate it to an ultimate pressure of 1 × 10^−6^ Pa using the turbo-molecular pump. Subsequently, we activate the NEG pump; Then we complete the NEG activation. After that, we cut the oxygen-free copper tubes to isolate the vacuum chamber from the turbo-molecular pump. Combined pumping stage: We operate the ion pump and the NEG pump collaboratively to improve the system vacuum to 1 × 10^−8^ Pa. Passive maintenance stage: The ion pump was shut off, relying solely on the NEG to maintain the vacuum environment, with a monitoring period of 35 days. Experimental data (Fig. 8b) and (Fig. 8c) indicate that after active pumping ceases, the vacuum pressure rises from 1 × 10^−8^ Pa to 2.8 × 10^−7^ Pa within 5 minutes, and then gradually increases to 4 × 10^−7^ Pa over the following 35 days. These results verify the high-efficiency adsorption capacity of NEGs for reactive gases under passive vacuum conditions, with the passive adsorption process effectively slowing the degradation trend of the chamber pressure. The accumulation phase of inert gas permeation occurred throughout the entire experiment. Helium permeation through the quartz window (permeability: 1.14 × 10⁻10^−10^ m^2^/s) is the dominant factor responsible for the slow degradation of vacuum, with a deterioration rate of 1.4 × 10^−10^ Pa/h. This process also includes contributions from vacuum leakage and material outgassing. We reconnect a quadrupole mass spectrometer to the system, as shown in (Fig. 9), to analyze the partial pressures of various residual gases under ultra-high vacuum. We find that the partial pressures of water vapor, oxygen, and hydrogen decrease continuously during pumping as the vacuum level improves, while the helium partial pressure shows a clear increasing trend. The minor fluctuations in vacuum pressure shown in the figure may also be influenced by the ambient laboratory temperature. By quantifying the pressure evolution patterns of passive vacuum systems, we provide solid experimental evidence and practical design guidelines for stable vacuum encapsulation technology suitable for space-borne quantum sensing applications.

**Fig. 10:**
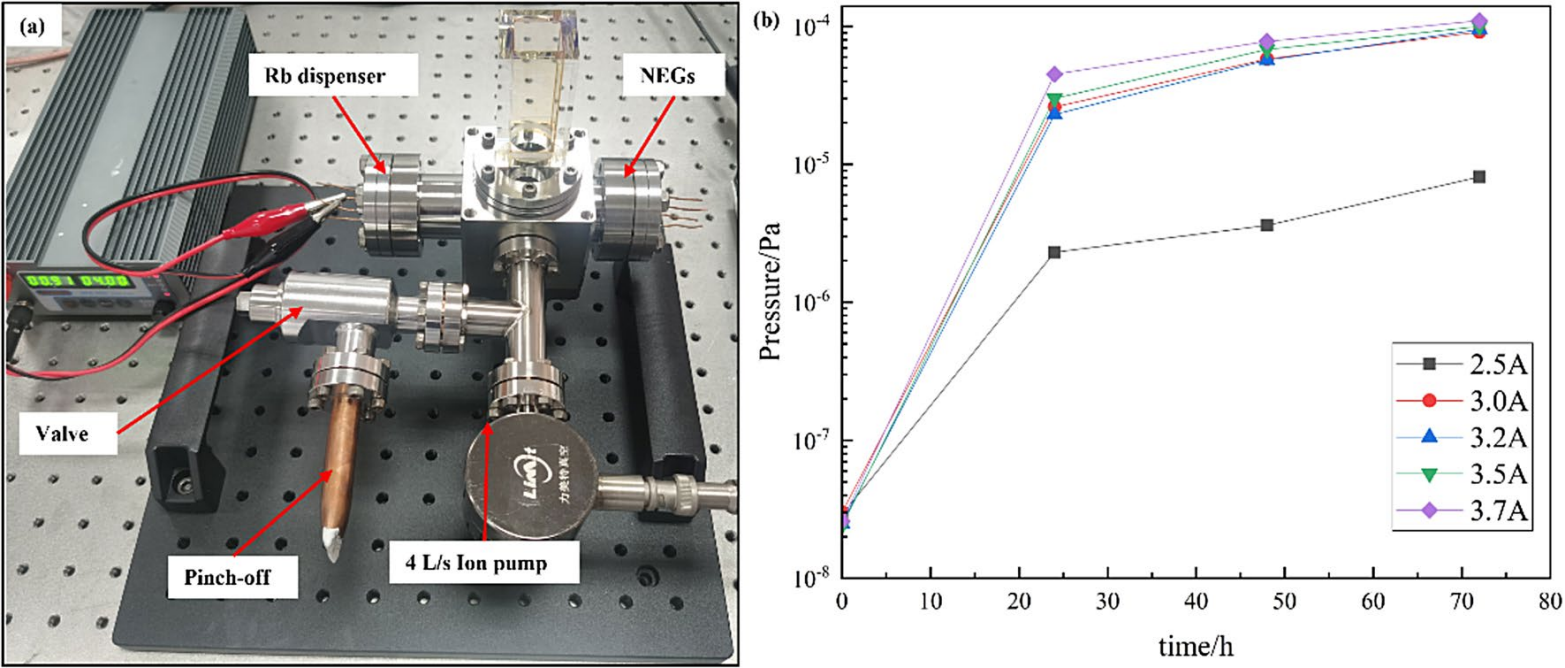
(**a**) Compact GMOT cold atom vacuum system. (**b**) Demonstration experiment of passive maintenance based on GMOT Cold Atom Vacuum System.

We designed a novel compact GMOT cold atom vacuum system (Fig. 10a) based on the above test results. The system comprises a compact titanium vacuum enclosure, a quartz glass chamber, two SAES non-evaporable getters (model ST171 HI/9.5-7.5), a compact ion pump with a pumping speed of 4 L/s, and three SAES rubidium dispensers. The optical path and magnetic field coils are yet to be installed, and the grating chip is not presented here. It will be mounted on top of the glass chamber. To facilitate grating installation, this particular window is only 2 mm thick, whereas all other glass windows have a thickness of 6 mm. The vacuum reading from the ion pump controller currently falls below 2.1 × 10^−9^ Pa. A simplified demonstration experiment involved applying different operating currents to the dispenser while simultaneously shutting down the ion pump to rely solely on the getter for vacuum maintenance. The time intervals were 24h, 48h, and 72 hours. The ion pump is then reactivated to record the instantaneous vacuum pressure. The experimental process and results are shown in Fig. 10(b). Under passive vacuum conditions, with an operating current applied to the dispenser continuously for 3 days, the vacuum pressure exhibits a positive correlation with both the magnitude of the dispenser current and the operating time, varying within the range of 8 × 10^−6^ Pa to 1 × 10^−4^ Pa

Although this method of measuring vacuum pressure by turning on the ion pump has certain errors, because the ion pump will exert a pumping effect once activated, the actual vacuum pressure should be lower. For the current non-purely passive vacuum device, this method can be used to estimate the pressure of the passive vacuum system in our setup when no vacuum gauge is installed.

### Miniaturized passive vacuum system for cold atoms on chip

**Fig. 11:**
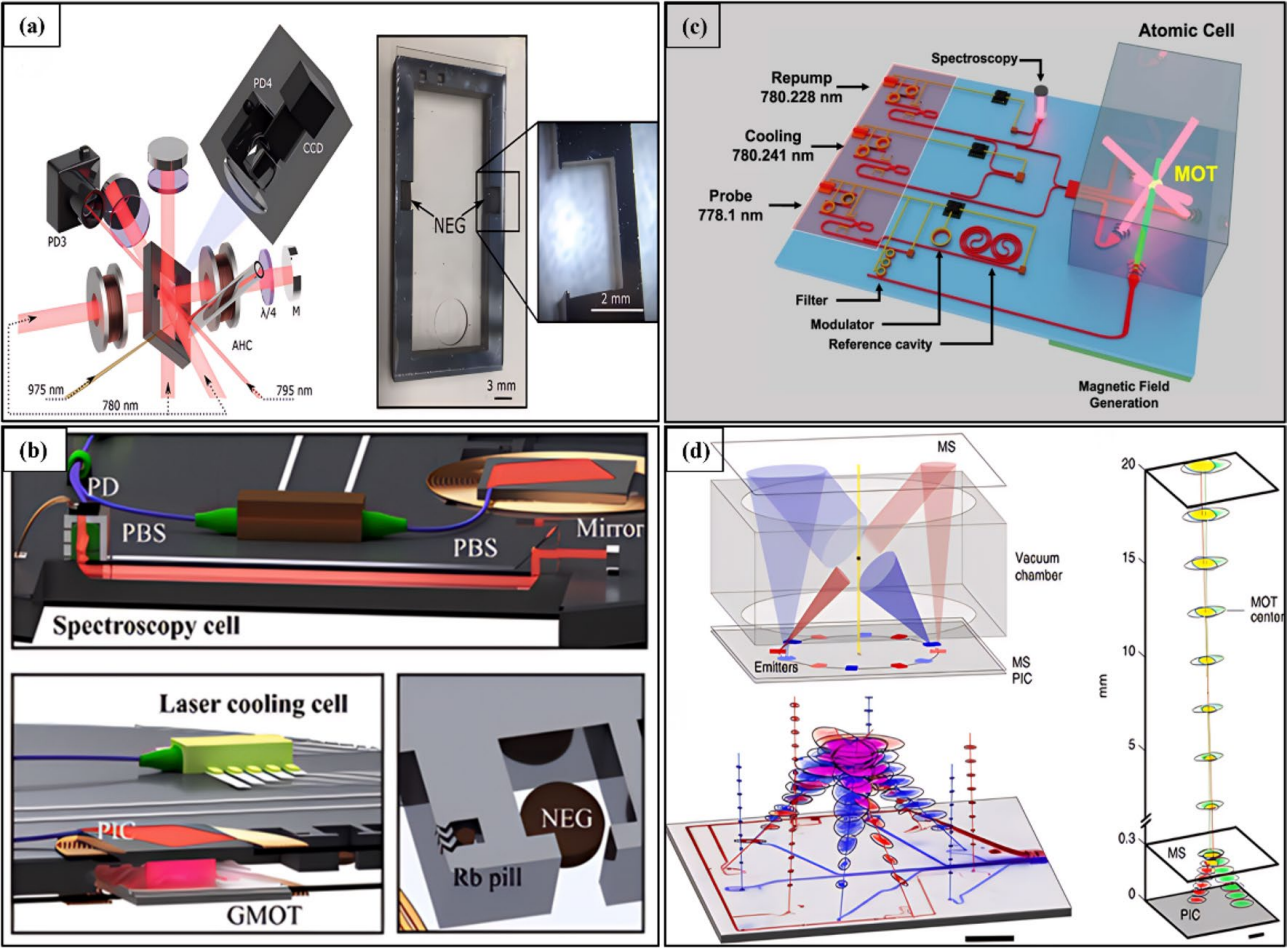
(**a**) A silicon-glass micro-fabricated UHV chamber embedded with a silicon-etched NEG chamber and ASG windows demonstrated the ability to repeatedly load a MOT within 10 minutes using a single laser-activated NEG.

A series of studies on on-chip cold atom sensors^110,111^ indicates that passive vacuum technology may be more suitable for ultra-high vacuum systems requiring on-chip micro-integration. A recent study^112^ of particular interest focused on the effect of NEGs on extending the observation time of laser-cooled atoms in MOTs. The investigation monitored changes in the number of atoms within the MOT, the rubidium vapor pressure, and variations in the non-rubidium background pressure before and after NEG activation. In a series of experiments, silicon-etched NEG cavities with aluminosilicate glass (ASG) windows were embedded into silicon-glass microfabricated UHV chambers (Fig. 11a). Results demonstrated that a single laser-activated NEG could repeatedly load the MOT within 10 minutes. Subsequent experiments investigated the ability to sustain the MOT solely through passive pumping within a borosilicate glass chamber containing five NEG elements. Results demonstrated that the MOT remained visible for over 4 days without an external active pumping system. While these vacuum systems retained ion pump interfaces and did not achieve fully passive maintenance, these validations hold critical value for applying passive vacuum techniques in developing portable, on-chip micro-cold-atom devices.

Research conducted in 2021^113^ proposed a chip-scale laser cooling platform solution. This platform, based on microfabricated optical components and vacuum devices (Fig. 11b), aims to establish a laser cooling system that combines low cost with mass production capability. The platform employs a 4-inch wafer stacking structure, with the vacuum chamber formed as a glass-silicon-glass sandwich using anodic bonding technology. This platform can integrate fiber-coupled photonic integrated circuits (PICs) (Fig. 11c) to guide all beams into the vacuum cavity. Recent studies have explored planar optics for chip-level incident light generation, shaping, and guidance: Firstly, light sources are injected into nanophotonic waveguides etched onto silicon nitride (SiN) substrates^114^ (this technique has been demonstrated to be compatible with MEMS vapor chambers). The PICs guide light into a SiN planar mode, which is then coupled to a tapered diffractive grating, ultimately outputting a collimated Gaussian mode into free space. Another noteworthy study (Fig. 11d) combines PICs with metasurfaces (MS)^20^ to achieve multi-beam generation for a compact strontium atomic clock. This system implements a 3D-MOT within a vacuum cavity measuring 25 mm^3^ with transmission losses of approximately 10–15 dB. While these publications highlight photonic integrated circuits and novel diffractive optical elements, they do not provide concrete solutions for the miniaturization of vacuum systems in on-chip cold atom sensors.

In practice, achieving a vacuum on a chip presents two major challenges. The first is the alkali metal source solution mentioned earlier. As previously analyzed, including in existing research^115–117^, the optimal atomic source for chip-level cooling platforms is solid-state alkali metal pellets: chambers housing these pellets can be formed by etching silicon frameworks, with external activation achieved via laser heating. The second challenge is ensuring long-term stability for passive vacuum maintenance. While NEGs offer significant advantages over traditional ion pumps for sustaining micro-vacuum systems, their critical limitation is the inability to evacuate inert gases—a problem thoroughly reviewed earlier. This can be addressed by employing low-helium-permeability materials to block external inert gas infiltration.

## Performance evaluation of the passive vacuum system

Ionization pressure gauges are traditional tools for ultra-high vacuum pressure measurement^118^, but suffer from high power consumption and excessive space requirements. Another commonly used ultra-high vacuum pressure measurement device—the residual gas analyzer—faces similar limitations. An alternative approach involves utilizing ion pumps for vacuum pressure measurement^118,119^. Although ion pumps are primarily employed to maintain vacuum pressure, their ability to measure pump flow can also serve as a pressure indicator. However, the vacuum measurement accuracy of ion pumps is slightly inferior to that of ionization gauges. Their detection limit is constrained by leakage current, reaching a minimum of approximately 10^−7^ Pa. The relationship between current and pressure is complex and varies with pump design. For the reasons stated above, none of these are suitable for the passive vacuum systems discussed in this paper. While passive vacuum systems offer significant advantages in terms of cost, maintenance requirements, and operating conditions, it is important to note that they do not inherently possess pressure measurement capabilities. Numerous studies^27–31,51,120–134^ have deduced precise vacuum pressure values by investigating the relationship between the loading dynamics of atoms in cold atom sensors and the presence of background gas pressure.

The passive ultra-high vacuum chamber uses atomic loading parameters to calculate the vacuum pressure^131^. According to the trap loading dynamics:

23 $$\frac{dN}{{dt}} = R - \Gamma N$$

In the equation, *N* is the number of atoms in the MOT, *R* is the loading rate, $\Gamma = \gamma_{Rb} P_{Rb} + \gamma_{bk} P_{bk} + \Gamma_{0}$ is the total rate of atomic loss, which is proportional to the rubidium pressure $P_{Rb}$ and background pressure $P_{bk}$. This loss rate depends on the collision rate $\gamma_{Rb}$ with rubidium, the collision rate $\gamma_{bk}$ with other background gases under pressure, and the density-dependent factor $\Gamma_{0}$ of the internal collisions within the cold atomic cloud. In a typical MOT, the density of the trapped atoms is limited by the light scattering force; under this constant density limit, *γ* can be approximated as a constant, and an exponential loading curve can be obtained:

24 $$N = N_{0} \left( {1 - \exp \left( { - t/\tau } \right)} \right)$$

Among them, $N_{0} = R\tau$, which is the total number of atoms loaded within the time constant $\tau = 1/\Gamma$. *N* and *N*_*0*_ are measured by observing the fluorescence of the atoms. $\gamma_{Rb}$ can be used to estimate the background pressure in a typical UHV system^27^:

25 $$P_{vacuum} < \left( {2 \times 10^{ - 8} Torr \cdot s} \right)/\tau$$

The methods discussed above provide a resolution comparable to ion pump measurements. However, due to collisions between trapped atoms limiting their lifetime, their minimum pressure detection limit is approximately 10^−8^ Pa. Nevertheless, the vacuum metrology field generally considers the larger calibration uncertainty acceptable. Notably, ionization pressure gauges exhibit sensitivity differences of up to twofold for H₂ and N₂ gases, and as much as eightfold for He and Ar gases^118^. As demonstrated in^119^, significant sensitivity variations also exist within ion pumps. In the absence of residual gas composition data, these factors may introduce substantial uncertainty. Nevertheless, ionization gauges and ion pumps perform well in numerous applications. Recent studies indicate that MOT-based loading measurements can achieve approximately double the accuracy compared to conventional techniques^27^.

**Fig. 12:**
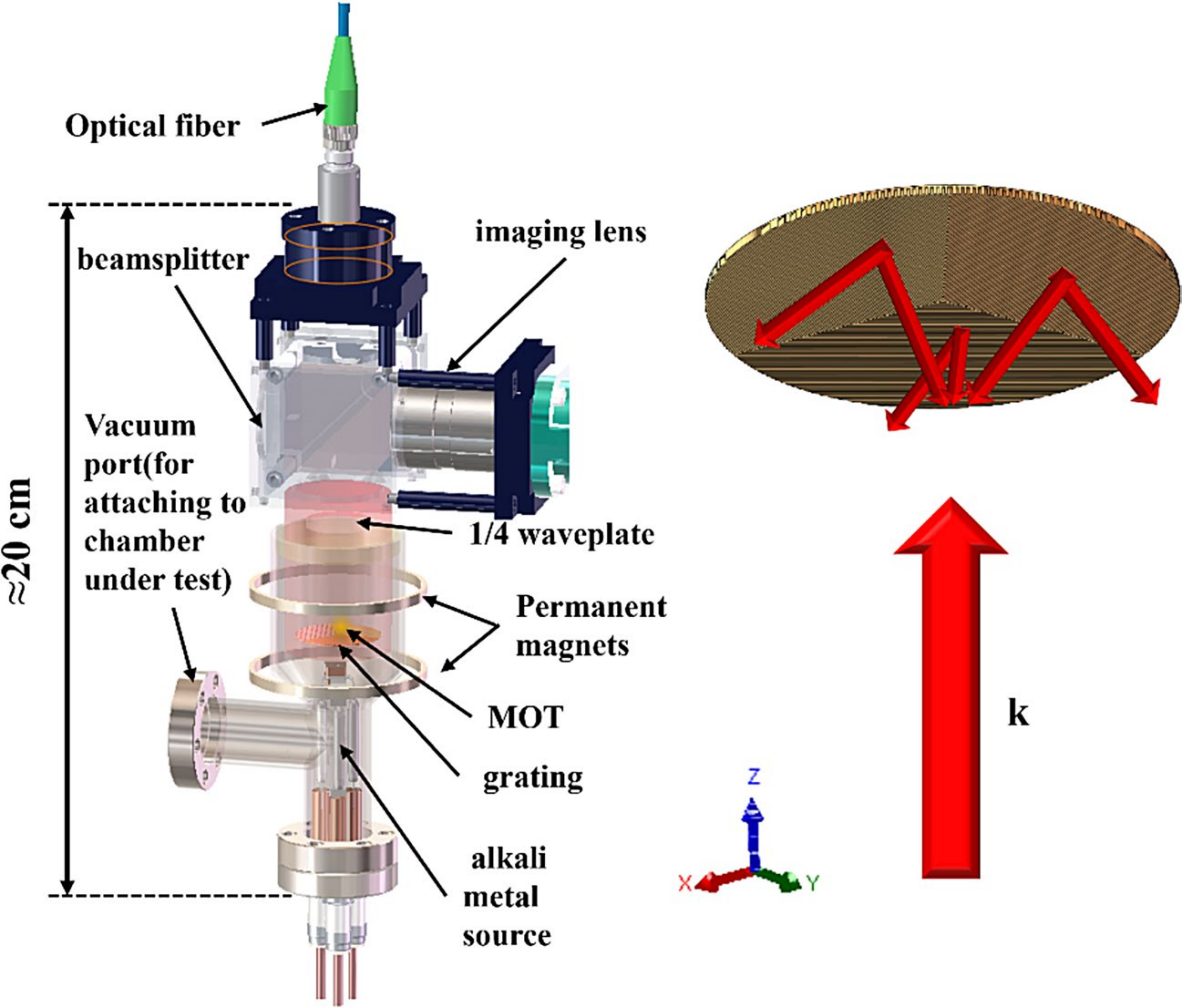
The schematic diagram of the “MOT vacuum gauge P-CAVS” is shown^28^. This design includes a MOT and a quadrupole magnetic trap, providing two operating modes. The expected corrections and related uncertainties under these two modes are analyzed, including atomic loss due to collisions, the fraction of excited states, and Majorana spin-flip losses.

Cold atom vacuum sensors (CAVS)^28^ have been identified as potential primary standards for pressure measurement in the UHV and XHV ranges. To realize deployable sensors within the “quantum SI” paradigm, miniaturization of cold atom technology is urgently needed to enable straightforward operation and integration with other vacuum chambers. Current efforts focus on developing laboratory-scale CAVS capable of cooling and trapping diverse sensing atoms, including ⁶Li, ⁷Li^131^, ⁸^5^Rb, and ⁸⁷Rb. However, background gas collisions represent a primary loss source in cold atom traps, necessitating precise correction and uncertainty assessment of this process. A recent related study has designed a portable cold atom vacuum scale (p-CAVS) based on microfabricated gratings (Fig. 12). This device adapts to existing vacuum gauge dimensions and provides precise pressure measurements. Its design integrates a magneto-optical trap with a quadrupole magnetic trap, enabling dual operating modes. Through detailed analysis of expected correction terms and associated uncertainties in both modes, a comprehensive uncertainty budget was established. This budget encompasses factors such as collision-induced atomic loss, excited-state fraction, and Majorana spin-flip loss, covering both Class B systematic errors and Class A random errors.

## Discussion and future prospects

The miniaturization of cold-atom platforms^135,136^ is crucial for advancing quantum technologies in various fields such as navigation, geological exploration, communication, and precision timing. Developing a fully integrated cold-atom measurement platform is essential to transition from laboratory experiments to practical applications. This endeavor involves downsizing vacuum systems, integrating laser systems, optimizing magneto-optical traps, enhancing ultracold atom sources, upgrading laser and optical systems for atomic interferometers, and improving detection techniques. Such a platform facilitates precise inertial measurements in space, including acceleration and rotation^137^.

MEMS ^138^ technology has enabled microfabrication and miniaturization, making chip-scale cold-atom vacuum systems feasible. Microfabrication techniques allow the production of chip-scale wavelength references for laser cooling, atom sources, and magnetic field generation. They also enable the miniaturization of vacuum chambers using materials like silicon, ceramics, and metal alloys^98^. This miniaturization trend aligns with the emergence of commercially available chip-scale atomic clocks (CSACs)^139^, which have reduced traditional bulky optical spectroscopy systems to sizes smaller than a grain of rice. Some initiatives have already commenced to miniaturize entire ultracold atom systems, exemplified by the backpack-sized iSense gravimeter^140^. However, achieving a level of complexity, size, and robustness comparable to CSACs will necessitate at least another decade of development.

## Data Availability

Data underlying the results presented in this paper are not publicly available at this time but may be obtained from the authors upon reasonable request.

## References

[1] Heavner, T. P. et al. NIST-F1: recent improvements and accuracy evaluations. *Metrologia* **42**, 411 (2005).

[2] Katori, H. Optical lattice clocks and quantum metrology. *Nat. Photonics* **5**, 203–210 (2011).

[3] Schioppo, M. et al. Ultrastable optical clock with two cold-atom ensembles. *Nat. Photonics* **11**, 48–52 (2017).

[4] Abend, S. et al. Technology roadmap for cold-atoms based quantum inertial sensor in space. *AVS Quantum Sci.*10.1116/5.0098119 (2023).

[5] Geiger, R. et al. High-accuracy inertial measurements with cold-atom sensors. *AVS Quantum Sci.*10.1116/5.0009093 (2020).

[6] Bidel, Y. et al. Absolute marine gravimetry with matter-wave interferometry. *Nat. Commun.*10.1038/s41467-018-03040-2 (2018). 29434193 10.1038/s41467-018-03040-2 PMC5809417

[7] Li, D. et al. Review of atom chips for absolute gravity sensors. *Sensors*10.3390/s23115089 (2023). 37299815 10.3390/s23115089 PMC10255705

[8] Ménoret, V. et al. Gravity measurements below 10−9g with a transportable absolute quantum gravimeter. *Sci. Rep.*10.1038/s41598-018-30608-1 (2018). 30120334 10.1038/s41598-018-30608-1 PMC6098009

[9] Vovrosh, J. et al. Advances in portable atom interferometry-based gravity sensing. *Sensors*10.3390/s23177651 (2023). 37688106 10.3390/s23177651 PMC10490657

[10] Weng, K. et al. Compact magneto-optical trap with a quartz vacuum chamber for miniature gravimeters. *J. Opt. Soc. Am. B* **37**, 1637–1642 (2020).

[11] Xu, R. et al. Magneto-optical traps for cold atomic gravimetry: Research status and development trends. *Appl. Sci. (Basel)*10.3390/app13106076 (2023).

[12] Bregazzi, A. et al. A cold-atom Ramsey clock with a low volume physics package. *Scientific Reports* **14**, 931 (2023). 10.1038/s41598-024-51418-8 PMC10776663 38195807

[13] Elvin, R. et al. Cold-atom clock based on a diffractive optic. *Opt. Express* **27**(26), 38359–38366 (2019). 31878604 10.1364/OE.378632

[14] Lewis, B. et al. A grating-chip atomic fountain. *Appl. Phys. Lett.* (2022).

[15] Schwindt, P. D. D. et al. A highly miniaturized vacuum package for a trapped ion atomic clock. *Rev. sci. instrum.* **87**, 053112 (2016). 27250397 10.1063/1.4948739

[16] Bongs, K. et al. Taking atom interferometric quantum sensors from the laboratory to real-world applications. *Nat. Rev. Phys.* **1**, 731–739 (2019).

[17] Barrett, B. et al. Mobile and Remote Inertial Sensing with Atom Interferometers. *arXiv: At. Phys.* **188**, 493–555 (2013).

[18] Kitching, J. E. et al. Atomic sensors – a review. *IEEE Sens. J.* **11**, 1749–1758 (2011).

[19] Isichenko, A. et al. Photonic integrated beam delivery for a rubidium 3D magneto-optical trap. *Nat. Commun.* **14**, 3080 (2023). 37248247 10.1038/s41467-023-38818-6 PMC10227028

[20] Ropp, C. et al. Integrating planar photonics for multi-beam generation and atomic clock packaging on chip. *Light Sci. Appl.* **12**, 83 (2023). 37009814 10.1038/s41377-023-01081-x PMC10068800

[21] Wang, Y. et al. The fabrication of MEMS alkali metal vapor cells based on ultrafast laser welding for single beam magnetometer. *Microsyst. Nanoeng.*10.1038/s41378-025-00976-6 (2025). 40813769 10.1038/s41378-025-00976-6 PMC12354740

[22] Li, J. et al. An integrated high-flux cold atomic beam source for strontium. *Rev. Sci. Instrum.*10.1063/5.0162128 (2023). 37695113 10.1063/5.0162128

[23] Lamporesi, G. et al. Compact high-flux source of cold sodium atoms. *Rev. Sci. Instrum.* **84**, 063102 (2013). 23822328 10.1063/1.4808375

[24] Firpo, G. & Pozzo, A. New getter pump for ultrahigh vacuum systems and transportable enclosure. *Rev. Sci. Instrum.* **75**, 4828–4832 (2004).

[25] Moore, K. L. et al. Collimated, single-pass atom source from a pulsed alkali metal dispenser for laser-cooling experiments. *Rev. Sci. Instrum.*10.1063/1.1841852 (2005).

[26] Scherer, D. R. et al. Characterization of alkali metal dispensers and non-evaporable getter pumps in ultrahigh vacuum systems for cold atomic sensors. *J. Vac. Sci. Technol.*10.1116/1.4757950 (2012).

[27] Arpornthip, T. et al. Vacuum-pressure measurement using a magneto-optical trap. *Phys. Rev. A* **85**, 033420 (2012).

[28] Eckel, S. P. et al. Challenges to miniaturizing cold atom technology for deployable vacuum metrology. *Metrologia* **55**, S182–S193 (2018). 10.1088/1681-7575/aadbe4 PMC6459404 30983635

[29] Moore, R. W. G. et al. Measurement of vacuum pressure with a magneto-optical trap: A pressure-rise method. *Rev. Sci. Instrum.*10.1063/1.4928154 (2015). 26429430 10.1063/1.4928154

[30] Zhang, X. et al. Vacuum pressure measurement of cold 7Li atoms in the magneto-optical and magnetic trap. *AIP Adv.*10.1063/5.0149991 (2023).

[31] Burrow, O. S. et al. A centilitre-scale vacuum chamber for compact ultracold quantum technologies. (2020).

[32] Lewin, G. & Marton, L. Fundamentals of vacuum science and technology. *Phys. Today* **18**, 76–78 (1965).

[33] Redhead, P. A. et al. *The physical basis of ultrahigh vacuum, High vacuum series* (Chapman and Hall, 1968).

[34] Perkins, W. G. Permeation and outgassing of vacuum materials. *J. Vac. Sci. Technol.* **10**, 543–556 (1973).

[35] De Segovia, J. L. Physics of outgassing. (1999).

[36] Norton, F. J. Helium diffusion through glass. *J. Am. Ceram. Soc.* **36**, 90–96 (1953).

[37] Shelby, J. E. Helium migration in natural and synthetic vitreous silica. *J. Am. Ceram. Soc.* **55**, 61–64 (1972).

[38] Dylla, H. F. et al. Correlation of outgassing of stainless steel and aluminum with various surface treatments. *J. Vac. Sci. Technol. A* **11**, 2623–2636 (1993).

[39] Jousten, K. THERMAL OUTGASSING. (1999).

[40] Dushman, S. et al. Scientific foundations of vacuum technique. *Am. J. of Phys.* **30**, 612–612 (1962).

[41] Takeda, M. et al. Hydrogen outgassing mechanism in titanium materials. *Appl. Surf. Sci.* **258**, 1405–1411 (2011).

[42] McConnon, A. Using passive pumping to make small vacuum systems that can sustain cold atoms for months. *Scilight*10.1063/10.0005673 (2021).

[43] Battes, K. et al. Outgassing rate measurements of stainless steel and polymers using the difference method. *J. Vac. Sci. Technol. A*10.1116/1.4905099 (2015).

[44] M. L. Stutzman, et al. A Comparison of Outgassing Measurements for Three Vacuum Chamber Materials. *AIP Conference Proceedings* 671, 300-306 (2003).

[45] Grangeon, F. et al. Development of an ultra-high vacuum system for space application. *Vacuum* **73**, 243–248 (2004).

[46] Watanabe, F. et al. Attaining an ultralow outgassing rate of 10–12 Pa m3 s−1 m−2 from an oxygen-free high conductivity copper chamber with beryllium–copper–alloy flanges. *J. Vac. Sci. Technol. A* **13**, 2587–2591 (1995).

[47] Kingery, W. D. and Bowen, H. K. Introduction to Ceramics (1976).

[48] Dellis, A. T. et al. Low helium permeation cells for atomic microsystems technology. *Opt. Lett.* **41**, 2775–2778 (2016). 27304286 10.1364/OL.41.002775

[49] Carlé, C. et al. On the reduction of gas permeation through the glass windows of micromachined vapor cells using Al2O3 coatings. *J. Appl. Phys.*10.1063/5.0213432 (2024).

[50] Vecchio, F. et al. Dispensing and hermetic sealing Rb in a miniature reference cell for integrated atomic clocks. *Procedia Eng.* **5**, 367–370 (2011).

[51] Burrow, O. S. et al. Stand-alone vacuum cell for compact ultracold quantum technologies. *Appl. Phys. Lett.*10.1063/5.0061010 (2021).

[52] Cooper, N. et al. Additively manufactured ultra-high vacuum chamber for portable quantum technologies. *Addit. Manuf.* **40**, 101898 (2021).

[53] Turnbull, J. C. Barium, strontium, and calcium as getters in electron tubes. *J. Vac. Sci. Technol.* **14**, 636–639 (1977).

[54] Benvenuti, C. et al. A novel route to extreme vacua: the non-evaporable getter thin film coatings. *Vacuum* **53**, 219–225 (1999).

[55] Maccallini, E.et al. Non evaporable getter (NEG) technology: A powerful tool for UHV-XHV systems. AIP Conference Proceedings 1451, 24-27 (2012).

[56] Manini, P. et al. A novel route to compact, high performance pumping in UHV–XHV vacuum systems. *Vacuum* **94**, 26–29 (2013).

[57] Ferrario, B. Chemical pumping in vacuum technology. *Vacuum* **47**, 363–370 (1996).

[58] Benvenuti, C. Influence of the substrate coating temperature on the vacuum properties of Ti–Zr–V non-evaporable getter films. *Vacuum* **71**, 307–315 (2003).

[59] Surendra, K. & Rao, A. D. P. Preparation and characteristics of Ti-Co / Zr-Co based NEG materials. *Hybrid Adv.* **1**, 100002 (2022).

[60] Chiggiato, P. & Pinto, P. C. Ti-Zr-V non-evaporable getter films: from development to large scale production for the Large Hadron Collider. *Thin Solid Films* **515**, 382–388 (2006).

[61] Jousten, K. *Handbook of Vacuum Technology* (Wiley, 2016).

[62] Benvenuti, C. et al. Decreasing surface outgassing by thin film getter coatings. *Vacuum* **50**, 57–63 (1998).

[63] Ferrario, B. et al. H 2 , D 2 AND T 2 PUMPING AND HANDLING IN FUSION EXPERIMENTS USING SORB-AC NON-EVAPORABLE GETTER CARTRIDGES. (1976).

[64] Benvenuti, C. & Francia, F. Room-temperature pumping characteristics of a Zr–Al nonevaporable getter for individual gases. *J. Vac. Sci. Technol. A* **6**, 2528–2534 (1988).

[65] Benvenuti, C. & Francia, F. Room temperature pumping characteristics for gas mixtures of a Zr–Al nonevaporable getter. *J. Vac. Sci. Technol. A* **8**, 3864–3869 (1990).

[66] Benvenuti, C. & Chiggiato, P. Pumping characteristics of the St707 nonevaporable getter (Zr 70 V 24.6-Fe 5.4 wt %). *J. Vac. Sci. Technol. A Vacuum Surf. Films* **14**, 3278–3282 (1996).

[67] Boffito, C. et al. A nonevaporable low temperature activatable getter material. *J. Vac. Sci. Technol.* **18**, 1117–1120 (1981).

[68] Barosi, A. & Giorgi, T. A. A non-evaporable getter for low temperatures. *Vacuum* **23**, 15–19 (1973).

[69] Yang, Y. et al. Activation of Zr, ZrVHf and TiZrV non-evaporative getters characterized by in situ synchrotron radiation photoemission spectroscopy. *Appl. Sci.* **11**, 4844 (2021).

[70] Benvenuti, C. et al. Vacuum properties of TiZrV non-evaporable getter films. *Vacuum* **60**, 57–65 (2001).

[71] Wang, S. et al. Activation characterization of the Ti-Zr-V getter films deposited by magnetron sputtering. *Appl. Surf. Sci.* **528**, 147059 (2020).

[72] Das, B. K. et al. Improvement of deuterium emission by St 172 NEG pump in a sealed off vacuum device. *Vacuum* **181**, 109743 (2020).

[73] Sartori, E. et al. Development of non evaporable getter pumps for large hydrogen throughput and capacity in high vacuum regimes. *Vacuum* **214**, 112198 (2023).

[74] Prodromides, A. et al. Lowering the activation temperature of TiZrV non- evaporable getter films. *Vacuum* **60**, 35–41 (2001).

[75] Prodromides, A. Non-evaporable getter thin film coatings for vacuum applications. (2002).

[76] Chuntonov, K. et al. New lithium gas sorbents: II. a mathematical model of the evaporation process. *J. Alloys Compd.* **456**, 187–193 (2008).

[77] Chuntonov, K. et al. New lithium gas sorbent: III. Experimental data on evaporation. *J. Alloys Compd.* **460**, 357–362 (2008).

[78] Kang, S. et al. Magneto-optic trap using a reversible, solid-state alkali-metal source,. *Opt. Lett.*10.1364/OL.44.003002 (2019). 31199366 10.1364/OL.44.003002 PMC11566723

[79] Bridge, E. M. et al. A vapor cell based on dispensers for laser spectroscopy,. *Rev. Sci. Instrum.*10.1063/1.3036980 (2009). 19191423 10.1063/1.3036980

[80] Little, B. J. et al. A passively pumped vacuum package sustaining cold atoms for more than 200 days. *AVS Quantum Sci.*10.1116/5.0053885 (2021).

[81] Bartalini, S. et al. Full characterization of the loading of a magneto–optical trap from an alkali metal dispenser. *Eur. Phys. J. D – At., Mol., Optical Plasma Phys.* **36**, 101–104 (2005).

[82] Griffin, P. F. et al. Fast switching of alkali atom dispensers using laser-induced heating. *Rev. Sci. Instrum.* **76**, 093102 (2005).

[83] Douahi, A. et al. Vapour microcell for chip scale atomic frequency standard. *Electron. Lett.* **43**, 279–280 (2007).

[84] McGilligan, J. P. et al. Dynamic Characterization of an Alkali-Ion Battery as a Source for Laser-Cooled Atoms. *Phys. Rev. A.* **13**, 044038 (2020).

[85] Berthoud, P. et al. A continuous beam of slow, cold cesium atoms magnetically extracted from a 2D magneto-optical trap. *Europhys. Lett.* **41**, 141 (1998).

[86] Fu, Z. et al. A new type of compact gravimeter for long-term absolute gravity monitoring. *Metrologia* **56**, 025001 (2019).

[87] Wu, B. et al. Construction of absolute gravity benchmark offshore with an atomic gravimeter. *IEEE Sens. J.* **24**, 23527–23536 (2024).

[88] Liew, L.-A. et al. Microfabricated alkali atom vapor cells. *Appl. Phys. Letters* **84**, 2694–2696 (2004).

[89] Radhakrishnan, S. and Lal, A. Alkali metal-wax micropackets for chip-scale atomic clocks. *The 13th International Conference on Solid-State Sensors, Actuators and Microsystems, 2005. Digest of Technical Papers. TRANSDUCERS '05.* **1**, 23-26 Vol. 21 (2005).

[90] Liew, L.-A. et al. Wafer-level filling of microfabricated atomic vapor cells based on thin-film deposition and photolysis of cesium azide. *Appl. Phys. Lett.* **90**, 114106 (2007).

[91] Gong, F. et al. Electrolytic fabrication of atomic clock cells. *2006 IEEE International Frequency Control Symposium and Exposition*. 711-714 (2006).

[92] Pelton, A. D. The Au−Rb (gold-rubidium) system. *Bull. Alloy Phase Diagr.* **7**, 139–142 (1986).

[93] Torralbo-Campo, L. et al. Light-induced atomic desorption in a compact system for ultracold atoms. Scientific Reports **5**, (2013). 10.1038/srep14729 PMC4602189 26458325

[94] Hatakeyama, A. et al. Classification of Light-Induced Desorption of Alkali Atoms in Glass Cells Used in Atomic Physics Experiments. *E-j. Surf. Sci. Nanotechnol.* **4**, 63–68 (2005).

[95] Du, S. et al. Atom chip bose-einstein condensation in a portable vacuum cell. *InternationalQuantum Electronics Conference, 2004. (IQEC)*. 1130-1131 (2004).

[96] Marinelli, C. et al. Desorption of Rb and Cs from PDMS induced by non resonant light scattering. *Eur. Phys. J. D – At., Mol., Optical Plasma Phys.* **37**, 319–325 (2006).

[97] Villalba, S. et al. Light-induced atomic desorption and diffusion of Rb from porous alumina. *Phys. Rev. A* **81**, 032901 (2010).

[98] McGilligan, J. P. et al. Micro-fabricated components for cold atom sensors,. *Rev. Sci. Instrum.* **93**(9), 091101 (2022). 36182455 10.1063/5.0101628

[99] McGilligan, J. P. et al. Phase-space properties of magneto-optical traps utilising micro-fabricated gratings. *Opt. Express* **23**(7), 8948–8959 (2015). 25968732 10.1364/OE.23.008948

[100] Barker, D. S. et al. Λ-enhanced gray molasses in a tetrahedral laser beam geometry. *Opt. Express* **30**(6), 9959–9970 (2021). 10.1364/OE.444711 PMC9843705 35299409

[101] Bregazzi, A. et al. Single-beam grating-chip 3D and 1D optical lattices. (2024). 10.1103/PhysRevLett.134.013001 39913708

[102] Hoth, G. W. et al. Atom number in magneto-optic traps with millimeter scale laser beams. *Opt. Lett.* **38**, 661–663 (2013). 23455257 10.1364/OL.38.000661

[103] Ohadi, H. et al. Magneto-optical trapping and background-free imaging for atoms near nanostructured surfaces. *Opt. Express* **17**, 23003–23009 (2009). 20052226 10.1364/OE.17.023003

[104] Nshii, C. C. et al. A surface-patterned chip as a strong source of ultracold atoms for quantum technologies. *Nat. Nanotechnol.* **8**(5), 321–324 (2013). 23563845 10.1038/nnano.2013.47

[105] Lee, J.et al. A Cold-Atom Interferometer with Microfabricated Gratings and a Single Seed Laser. (2021).

[106] Lee, J. et al. A compact cold-atom interferometer with a high data-rate grating magneto-optical trap and a photonic-integrated-circuit-compatible laser system. *Nat. Communi.* **13**, 5131 (2022). 10.1038/s41467-022-31410-4 PMC9436985 36050325

[107] Yu, Z.-G. et al. Low power consumption grating magneto-optical trap based on planar elements. *Optics express* **32**(6), 8919–8928 (2024). 38571137 10.1364/OE.518268

[108] Earl, L. et al. Demonstration of a compact magneto-optical trap on an unstaffed aerial vehicle. *Atoms*10.3390/atoms10010032 (2022).

[109] Yu, Z.-G. et al. Grating magneto-optical trap optimization and drift-mitigation based on Bayesian learning. *Applied Physics Letters.*10.1063/5.0187999 (2024).

[110] Bregazzi, A. et al. A simple imaging solution for chip-scale laser cooling. *Appl. Phys. Lett.*10.1063/5.0068725 (2021).

[111] Burrow, O. S. et al. Optimal binary gratings for multi-wavelength magneto-optical traps. *Opt. Express* **31**(24), 40871–40880 (2023). 38041377 10.1364/OE.498606

[112] Boudot, R. et al. Enhanced observation time of magneto-optical traps using micro-machined non-evaporable getter pumps. *Sci. Rep.*10.1038/s41598-020-73605-z (2020). 33024172 10.1038/s41598-020-73605-z PMC7538997

[113] Bregazzi, A. et al. Enabling the mass production of a chip-scale laser cooling platform. In *Other Conferences*. (2021).

[114] Isichenko, A. et al. Photonic integrated beam delivery for a rubidium 3D magneto-optical trap. *Nat. Commun.* **14**, 3080 (2023). 37248247 10.1038/s41467-023-38818-6 PMC10227028

[115] Hummon, M. T. et al. Photonic chip for laser stabilization to an atomic vapor with 10−11 instability. *Optica*10.1364/OPTICA.5.000443 (2018).

[116] Newman, Z. L. et al. Architecture for the photonic integration of an optical atomic clock. *Optica*10.1364/OPTICA.6.000680 (2019).

[117] Stern, L. et al. Chip-scale atomic diffractive optical elements. *Nat. Commun.* **10**, 3156 (2019). 31316075 10.1038/s41467-019-11145-5 PMC6637105

[118] Lafferty, J. M. & Rubin, L. G. Foundations of Vacuum Science and Technology. *Phys. Today* **52**, 86–88 (1999).

[119] Welch, K. M. Capture pumping technology. (2001).

[120] Aruga, T. & Murata, Y. Alkali-metal adsorption on metals. *Prog. Surf. Sci.* **31**, 61–130 (1989).

[121] Bali, S. et al. Quantum-diffractive background gas collisions in atom-trap heating and loss. *Phys. Rev. A* **60**, R29–R32 (1999).

[122] Bjorkholm, J. E. Collision-limited lifetimes of atom traps. *Phys. Rev. A* **38**, 1599–1600 (1988). 10.1103/physreva.38.1599 9900540

[123] Fagnan, D. E. et al. Observation of quantum diffractive collisions using shallow atomic traps. *Phys. Rev. A* **80**, 022712 (2009).

[124] Garofalini, S. H. Molecular dynamics computer simulations of silica surface structure and adsorption of water molecules. *J. Non-Cryst. Solids* **120**, 1–12 (1990).

[125] Gensemer, S. D. et al. Trap-loss collisions of ${}^{85}\mathrm{Rb}$ and ${}^{87}\mathrm{Rb}:$ Dependence on trap parameters. *Phys. Rev. A* **56**, 4055–4063 (1997).

[126] Margenau, H. & Kestner, N. R. Preface to the Second Edition. In *Theory of Intermolecular Forces* 2nd edn (eds Margenau, H. & Kestner, N. R.) 11 (Pergamon, 1969).

[127] Miller, T. M. & Bederson, B. Atomic and molecular polarizabilities-A review of recent advances. In *Advances in Atomic And Molecular Physics* (eds Bates, D. R. & Bederson, B.) 1–55 (Academic Press, 1978).

[128] Prentiss, M. et al. Atomic-density-dependent losses in an optical trap. *Optics Letters* **13**, 452–454 (1988). 19745929 10.1364/ol.13.000452

[129] Raab, E. L. et al. Trapping of neutral sodium atoms with radiation pressure. *Phys. Rev. Letters* **59**, 2631–2634 (1987). 10.1103/PhysRevLett.59.2631 10035608

[130] Sesko, D. W. et al. Behavior of neutral atoms in a spontaneous force trap. *J. Opt. Soc. Am. B Opt. Phys.* **8**, 946–958 (1991).

[131] Sun, W. et al. Cold atom technology applied to ultra-high vacuum (UHV) measurements. *Vacuum* **222**, 113079 (2024).

[132] Supakar, S. et al. Development of a pyramidal magneto-optical trap for pressure sensing application. *Optical Eng.* **63**, 054103–054103 (2023).

[133] Van Dongen, J. et al. Trap-depth determination from residual gas collisions. *Phys. Rev. A* **84**, 022708 (2011).

[134] Zhang, S.-Z. et al. Vacuum pressure measurement based on 6Li cold atoms in a magneto-optical trap. *Acta Physica Sinica* **71**, 145–152 (2022).

[135] McGilligan, J. P.et al. Deep silicon cell fabrication for chip-scale atomic sensors. In *Quantum West*. (2023).

[136] Dyer, S. D. et al. A tuneable wavelength reference for chip-scale laser cooling. (2022).

[137] McGilligan, J. P. et al. Progress towards a fully integrated cold-atom measurement platform. In *OPTO*. (2022).

[138] Liew, L.-A. et al. Microfabricated alkali atom vapor cells. *Appl. Phys. Lett.* **84**, 2694–2696 (2004).

[139] Symmetricom SA.45 chip scale atomic clock. (2011).

[140] de Angelis, M. et al. iSense: A portable ultracold-atom-based gravimeter. *Procedia Comput. Sci.* **7**, 334–336 (2011).

